# Single‐cell RNA sequencing reveals immune cell dysfunction in the peripheral blood of patients with highly aggressive gastric cancer

**DOI:** 10.1111/cpr.13591

**Published:** 2024-02-06

**Authors:** Rui Ma, Xuemeng Zhou, Xiaohui Zhai, Chuyue Wang, Rong Hu, You Chen, Liyang Shi, Xing Fang, Yuan Liao, Lifeng Ma, Mengmeng Jiang, Junqing Wu, Renying Wang, Jiao Chen, Taiyuan Cao, Ge Du, Yingying Zhao, Weili Wu, Haide Chen, Shanshan Li, Qizhou Lian, Guoji Guo, Jian Xiao, Andrew P. Hutchins, Ping Yuan

**Affiliations:** ^1^ Guangdong Institute of Gastroenterology Guangzhou China; ^2^ Guangdong Provincial Key Laboratory of Colorectal and Pelvic Floor Disease The Sixth Affiliated Hospital, Sun Yat‐sen University Guangzhou China; ^3^ Shenzhen Key Laboratory of Gene Regulation and Systems Biology, Department of Biology School of Life Sciences, Southern University of Science and Technology Shenzhen China; ^4^ Department of Medical Oncology The Sixth Affiliated Hospital, Sun Yat‐sen University Guangzhou China; ^5^ Center for Stem Cell and Regenerative Medicine, and Bone Marrow Transplantation Center of the First Affiliated Hospital Zhejiang University School of Medicine Hangzhou China; ^6^ Zhejiang Provincial Key Lab for Tissue Engineering and Regenerative Medicine Dr. Li Dak Sum & Yip Yio Chin Center for Stem Cell and Regenerative Medicine Hangzhou China; ^7^ Liangzhu Laboratory Zhejiang University Medical Center Hangzhou China; ^8^ Faculty of Synthetic Biology Shenzhen Institute of Advanced Technology, Chinese Academy of Sciences Shenzhen China; ^9^ Guangzhou Institute of Eugenics and Perinatology, Guangzhou Women and Children's Medical Center Guangzhou Medical University Guangzhou China; ^10^ Institute of Hematology Zhejiang University Hangzhou China; ^11^ Department of Medical Oncology Guangdong Provincial People's Hospital, Guangdong Academy of Medical Sciences, Southern Medical University Guangzhou China; ^12^ Department of General Surgery The Sixth Affiliated Hospital, Sun Yat‐sen University Guangzhou China; ^13^ Biomedical Innovation Center The Sixth Affiliated Hospital, Sun Yat‐sen University Guangzhou China

## Abstract

Highly aggressive gastric cancer (HAGC) is a gastric cancer characterized by bone marrow metastasis and disseminated intravascular coagulation (DIC). Information about the disease is limited. Here we employed single‐cell RNA sequencing to investigate peripheral blood mononuclear cells (PBMCs), aiming to unravel the immune response of patients toward HAGC. PBMCs from seven HAGC patients, six normal advanced gastric cancer (NAGC) patients, and five healthy individuals were analysed by single‐cell RNA sequencing. The expression of genes of interest was validated by bulk RNA‐sequencing and ELISA. We found a massive expansion of neutrophils in PBMCs of HAGC. These neutrophils are activated, but immature. Besides, mononuclear phagocytes exhibited an M2‐like signature and T cells were suppressed and reduced in number. Analysis of cell‐cell crosstalk revealed that several signalling pathways involved in neutrophil to T‐cell suppression including APP‐CD74, MIF‐(CD74+CXCR2), and MIF‐(CD74+CD44) pathways were increased in HAGC. NETosis‐associated genes S100A8 and S100A9 as well as VEGF, PDGF, FGF, and NOTCH signalling that contribute to DIC development were upregulated in HAGC too. This study reveals significant changes in the distribution and interactions of the PBMC subsets and provides valuable insight into the immune response in patients with HAGC. S100A8 and S100A9 are highly expressed in HAGC neutrophils, suggesting their potential to be used as novel diagnostic and therapeutic targets for HAGC.

## Graphical abstract

Highly aggressive gastric cancer (HAGC) is a gastric cancer characterized by bone marrow metastasis and disseminated intravascular coagulation (DIC), which has a very poor prognosis. Peripheral blood mononuclear cells (PBMCs) were collected from seven HAGC patients, six normal advanced gastric cancer (NAGC) patients, and five healthy individuals for single‐cell RNA sequencing using the microwell‐seq platform. We identified the cell subpopulations and found that in HAGC patient blood immature neutrophils were greatly increased. Mononuclear phagocytes showed M2‐like signatures and T cells were suppressed and reduced in number. Together, they contributed to an immunosuppressive environment in HAGC patients. We also investigated the crosstalk among PBMCs and revealed that intercellular communications were greatly increased in HAGC. Besides, S100A8/A9 and multiple signalling pathways that contribute to DIC were upregulated in HAGC, suggesting their role in HAGC development.

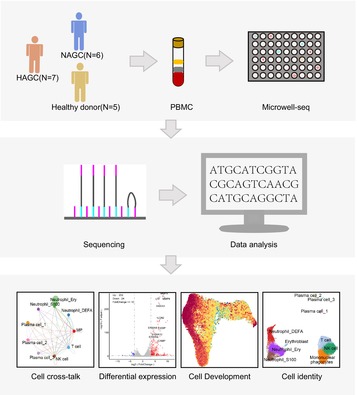

## 1 INTRODUCTION

Gastric cancer (GC) is the fifth most prevalent cancer worldwide, with a notably high incidence in East Asia.^1^ In certain instances, GC is presented with bone marrow metastasis (BMM) and disseminated intravascular coagulation (DIC), and is diagnosed as highly aggressive GC (HAGC). In contrast to normal advanced gastric cancer (NAGC) which lacks DIC, HAGC has a very poor prognosis and is relatively under‐studied.^2^

DIC is the primary clinical symptom and the main cause of fatality in patients with HAGC.^2^ DIC is a clinicopathological syndrome that arises in multiple diseases due to coagulation dysfunction. In cancer patients, tissue necrosis resulting from malignant solid tumours or their contiguous tissues can induce the release of diverse molecular factors, such as cancer procoagulant and tissue factor, which promote blood coagulation and disrupt the balance between coagulation and anticoagulation, resulting in DIC.^3^ Additionally, blood abnormalities have also been implicated in the pathogenesis of DIC. Damage to mononuclear phagocytes responsible for phagocytosing or clearing procoagulant substances contributes to DIC.^4^ Neutrophil dysfunction also plays a significant role in DIC. Upon stimulation, neutrophils release nuclear components into the extracellular matrix where they form a net‐like structure called the neutrophil extracellular traps (NETs). NETs can act as scaffolds to retain and activate platelets, which lead to thrombus formation and DIC.^5^ Nevertheless, the etiology of DIC in patients with HAGC remains unclear.

Tumour cells exert a significant influence on the immune system of patients, and peripheral blood cells can mediate the systemic immune response to tumour development.^6^ Research indicates that changes in peripheral blood mononuclear cells (PBMCs) are associated with the progression of tumour malignancy and patient prognosis.^6^ Therefore, PBMCs can potentially help assess the disease progression and immune status of patients. In order to avoid subjecting HAGC patients, who are often in poor physical condition, to undue harm, we chose to focus solely on collecting blood and studying PBMCs to gain insight into the pathogenesis of HAGC.

HAGC is highly heterogeneous and therapeutically challenging. While traditional bulk RNA‐seq may offer insights into macro‐level gene expression from tens of thousands of cells in samples, it does not allow us to account for each individual cell's effects, thus losing critical data. This problem can be solved by single‐cell RNA sequencing, which has yielded significant insights into various types of gastric cancer.^7^, ^8^, ^9^, ^10^ Using single‐cell sequencing technology that provides unparalleled depth and precision, we may be able to discover new diagnostic and treatment targets for HAGC.

Here, using the microwell‐seq platform,^11^, ^12^ we constructed a high‐quality single‐cell atlas of PBMCs from HAGC patients and NAGC patients, as well as healthy individuals. We demonstrated that HAGC was associated with substantial changes in the composition and activity of PBMCs. The neutrophil population was significantly increased in HAGC with a preponderance of immature and activated cells. Mononuclear phagocytes (MPs) showed signs of bias toward an M2‐like immunosuppressive signature. Conversely, T/NK cells displayed signs of suppression of activation, although not exhaustion. Additionally, our data suggested several signalling pathways that mediate the suppression of T cells, including APP‐CD74, MIF‐(CD74+CXCR2), and MIF‐(CD74+CD44) in HAGC. Meanwhile, S100A8 and S100A9 as well as VEGF, PDGF, FGF, and NOTCH signalling pathways that are implicated in the development of DIC were highly upregulated in HAGC neutrophils. Our study highlights significant alterations in the distribution and interactions of PBMC subsets in HAGC, reflecting a substantial remodelling of the patient's immune landscapes. This information offers a novel theoretical framework that could provide valuable insights into the precise diagnosis and treatment of HAGC, representing a significant advancement in our understanding of this disease.

## 2 RESULTS

### 2.1 Single‐cell RNA sequencing of PBMCs from HAGC patients reveals disturbances in cell type composition

**FIGURE 1:**
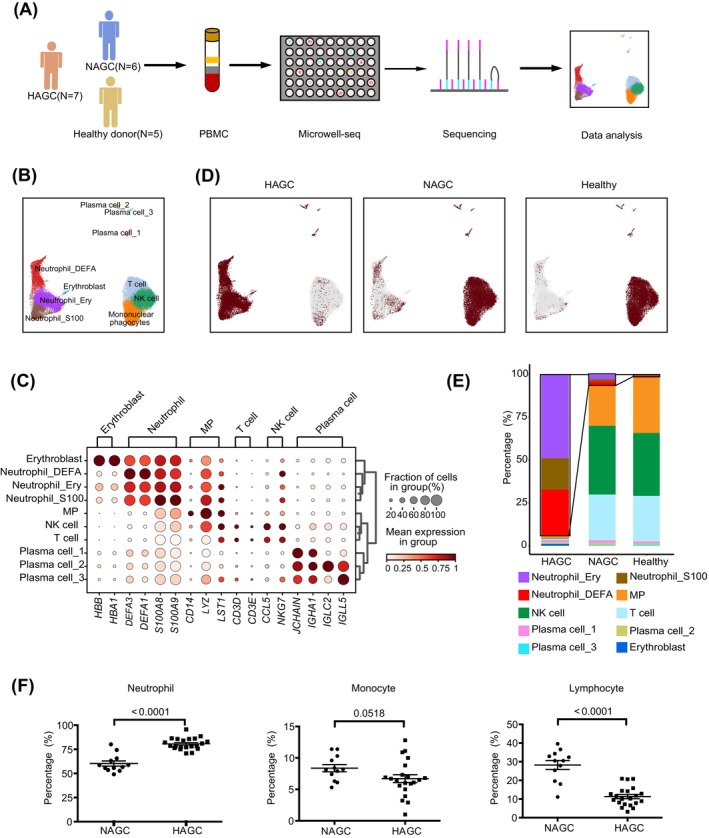
Single‐cell RNA‐seq analysis reveals abnormal cell type compositions in PBMCs from HAGC patients. (A) Study design overview: PBMCs were collected from patients with HAGC (*n* = 7), NAGC (*n* = 6), and healthy controls (*n* = 5). PBMCs were subjected to the microwell‐seq platform for scRNA‐seq library preparation. (B) UMAP visualization of 51,631 single cells for all combined samples. Cells were clustered using the Leiden algorithm (resolution = 0.9). (C) Bubble plot illustrating selected marker genes for each cluster. The color scheme represents the scaled mean expression from (0 = white, 1 = red) in the group. The bubble size indicates the fraction of cells expressing marker genes in the group. (D) Split UMAP distribution displaying the clusters in PBMCs and coloured by the sample origin. (E) Scaled bar chart showing the proportion of cell types in each sample. (F) Clinical blood test data plots representing the absolute numbers (top) and fractions (bottom) of neutrophils (left panel), monocytes (middle panel), and lymphocytes (right panel) in HAGC and NAGC patients. Each dot represents a single patient. The detailed data are listed in Table S1. And statistical significance was determined using two‐sided Mann‐Whitney U test.

A total of 18 peripheral blood samples were collected from seven HAGC patients, six NAGC patients and five healthy individuals for single‐cell RNA sequencing using microwell‐seq platform (Figure 1A and Table S1).

In total, 51,631 PBMCs passed the quality control, and each cell expressed approximately 469 genes with a mean read count of 934 (Supplementary Figure S1). A roughly equivalent contribution from the three conditions to the final number of cells was observed (Supplementary Figure S1). We projected the resulting PBMC gene expression using UMAP and identified clusters corresponding to typical PBMC cell types (Figure 1B). The identified cell types included mononuclear phagocytes (MPs), which highly express *CD14*, *LYZ* and *LST1*; T cells, which highly express *CD3D* and *CD3E*; NK cells, which highly express *CCL5* and *NKG7* and plasma cells (B cells), which highly express *JCHAIN*, *IGHA1*, *IGLC2* and *IGLL5* (Figure 1B, C, Table S2 and S3). Three dominant subpopulations of neutrophils were identified based on their differential gene expression profiles, which included DEFA (high expression of *DEFA3* and *DEFA1*), S100 (high expression of *S100A8* and *S100A9*), and Ery‐like (high expression of *HBB* and *HBA1*) subpopulation (Figure 1B, C and Supplementary Figure S1). The presence of high levels of pro‐inflammatory factors‐defensins (DEFA1/3 genes) and S100 in neutrophils,^13^, ^14^, ^15^ suggests a strong pro‐inflammatory systemic response in patients with HAGC.

Comparing the PBMC compositions in NAGC patients and healthy individuals, PBMCs of HAGC patients did not exhibit any unique cell type. However, there were significant alterations in the proportion of PBMCs. HAGC patient samples showed a remarkable increase in neutrophils, whereas the proportions of MPs, T cells, and NK cells were significantly reduced as compared to NAGC patients and healthy individuals (Figure 1D, E and Supplementary Figure S1). These findings were consistent with the clinical blood test result, which demonstrated increased neutrophils and decreased lymphocytes in HAGC patients compared to NAGC patients (Figure 1F and Table S1). Additionally, the single‐cell RNA‐sequencing analysis showed approximately 10% neutrophils in NAGC PBMCs, whereas the clinical blood test reported around 60% neutrophils. This disparity was attributed to the fact that the clinical blood test encompasses all blood cells, whereas our single‐cell RNA‐sequencing specifically detects PBMCs with a single round nucleus. Hence, this finding suggests that approximately one‐sixth of the neutrophils in NAGC resemble the round nucleus neutrophils found in HAGC, whereas the remaining neutrophils in NAGC patients exhibit normal polymorphonuclear characteristics.

### 2.2 PBMCs of HAGC patients consist primarily of immature neutrophils

**FIGURE 2:**
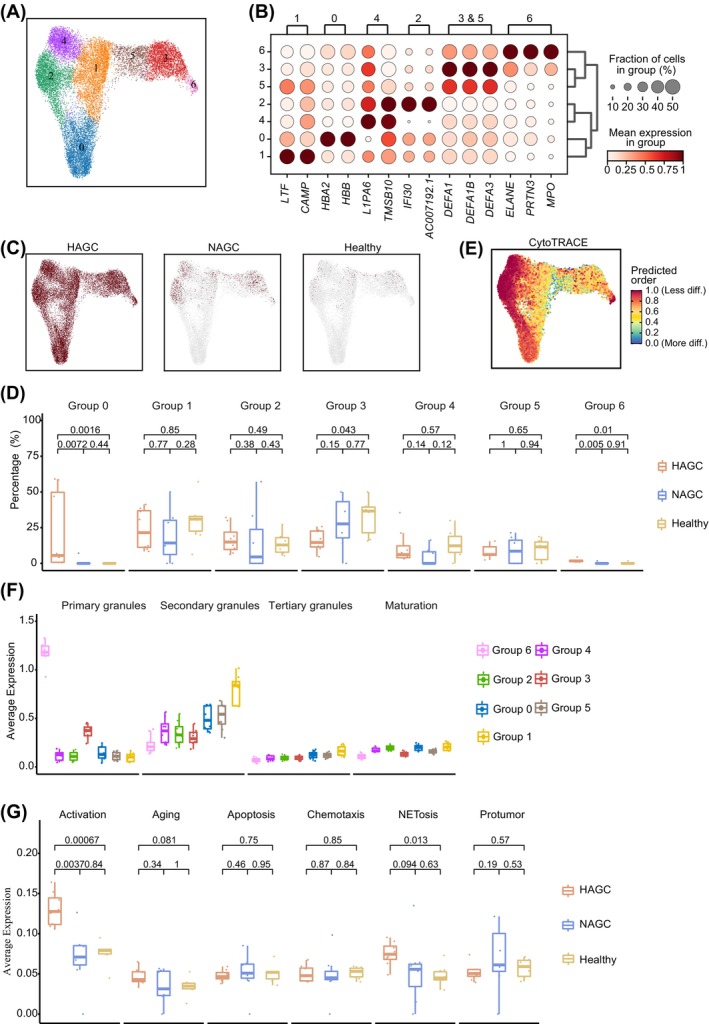
Immature neutrophils in PBMCs of HAGC patients. (A) UMAP visualization of the neutrophil subclusters after re‐clustering using the Leiden algorithm (resolution = 0.8). (B) Bubble plot displaying specific genes for each neutrophil category. The color scheme represents the scaled mean expression (0 = white, 1 = red) in the group. The bubble size indicates the fraction of cells expressing marker genes. (C) Split UMAP visualization of neutrophils, highlighting their origin (marked in red). (D) Boxplot demonstrating the proportions of neutrophil subtypes in each group. Statistical significance was determined using a two‐sided unpaired Welch's *t*‐test. (E) UMAP visualization of neutrophils coloured according to CytoTRACE scores. The color scheme represents the predicted order of differentiation, with 1 (red) indicating less differentiation and 1 (blue) indicating more differentiation. (F) Boxplot showing primary, secondary and tertiary granule scores, as well as the maturation score based on the gene sets listed in Table S2 for neutrophil subclusters across different conditions. (G) Boxplot showing activation, aging, apoptosis, chemotaxis, NETosis and pro‐tumour score based on the gene sets outlined in Table S2 across different conditions for neutrophil subclusters. Statistical significance was calculated using a two‐sided unpaired Welch's *t*‐test.

As neutrophils expanded significantly in HAGC, we then isolated and re‐clustered the neutrophils from the PBMC data. This process identified seven new subclusters, with subclusters 3, 5, and 6 corresponding to DEFA‐expressing neutrophils; subclusters 0, 2, and 4 corresponding to Ery‐like neutrophils; and subcluster 1 corresponding to S100‐expressing neutrophils (Figure 2A, Supplementary Figure S2A and Table S4). Each sub‐cluster exhibited characteristic gene expression patterns. Subcluster 0 showed high expression of HBA2 and HBB, which are specific to the red blood cell lineage, while subcluster 1 showed high expression of LTF and CAMP. Subcluster 2 showed high expression of IFI30 and AC007192.1 l. Subclusters 3 and 5 showed high expression of DEFA1, DEFA1B and DEFA3. Subcluster 4 showed high expression of L1PA6 and TMSB10. Subcluster 6 showed high expression of ELANE, PRTN3 and MPO (Figure 2B and Supplementary Figure S2B). Interestingly, HAGC patients had a unique subcluster of neutrophils (subcluster 0). These cells expressed *HBA2* and *HBB*, which are normally associated with red blood cell development. Their expression in the neutrophils of HAGC patients might reflect the influence of metastatic cancer cells on the bone marrow microenvironment, leading to abnormal neutrophil development. Additionally, the proportion of neutrophils in subcluster 6 was significantly increased in HAGC patients compared to NAGC patients and healthy individuals, while the proportions of other subclusters remained relatively unchanged (Figure 2C, D). Neutrophils in subcluster 6 expressed NETosis genes such as *MPO*, *ELANE* and *PRTN3*, indicating increased neutrophil NET formation in HAGC patients.

Moreover, our investigation revealed that HAGC neutrophils expressed genes associated with the early stages of neutrophil differentiation,^16^ such as *LTF*, *PRTN3* and *ELANE* (Supplementary Figure S2B). This intriguing finding suggests the existence of potential maturation abnormalities in HAGC neutrophils. In order to delve deeper into this phenomenon, we employed CytoTRACE to estimate the pseudo‐time trajectory of neutrophils.^17^ The analysis results revealed that clusters 2, 6, and 4 displayed lesser degrees of differentiation, while clusters 0 and 1 demonstrated intermediate differentiation, and clusters 5 and 3 exhibited more advanced differentiation (Figure 2E and Supplementary Figure S2C). Additional support for these observations was obtained through trajectory analysis using scVelo,^18^ which further confirmed the lower differentiation status of cells from clusters 2 and 4 (Supplementary Figure S2D). To gain more insights into the maturation issue, we compared HAGC neutrophils with bone marrow neutrophils of healthy individuals.^19^ Interestingly, CytoTRACE revealed that HAGC neutrophils exhibited lower levels of differentiation when compared to fully developed neutrophils in the bone marrow (Supplementary Figure S2E, F). In addition, representative granule genes are expressed during the process of neutrophil maturation.^20^ Therefore, we also investigated the expression of granule genes in each sub‐cluster of HAGC neutrophils. Notably, sub‐cluster 6 exhibited high expression of primary granule genes, whereas sub‐clusters 0 and 1 displayed increased expression of secondary granule genes. The expression of tertiary and mature granule genes was quite low across all sub‐clusters, indicating the immaturity of overall HAGC neutrophils (Figure 2F; Supplementary Figure S2G).

Next, we examined gene sets related to various biological categories, including activation, aging, apoptosis, chemotaxis, NETosis, and pro‐tumour responses in neutrophils.^16^, ^21^, ^22^ Interestingly, we observed that only genes associated with activation and NETosis were significantly upregulated in HAGC neutrophils (Figure 2G and Supplementary Figure S2H). Collectively, these results suggest that HAGC is characterized by a significant expansion of the neutrophils, which, although partially activated, retain an immature state. Furthermore, since NETs are involved in the pathophysiology of DIC, upregulation of NETosis‐associated genes in neutrophils may contribute to DIC in patients with HAGC.

### 2.3 Mononuclear phagocytes of HAGC present an M2‐like polarization signature

In addition to neutrophils, MPs within myeloid cells also play influential roles in response to tumour.^23^ Notably, the MPs in HAGC patients did not form any discrete clusters. However, they constituted only a minor fraction of the total PBMCs when compared to MPs from NAGC patients and healthy individuals (Figure 1D, E).

**FIGURE 3:**
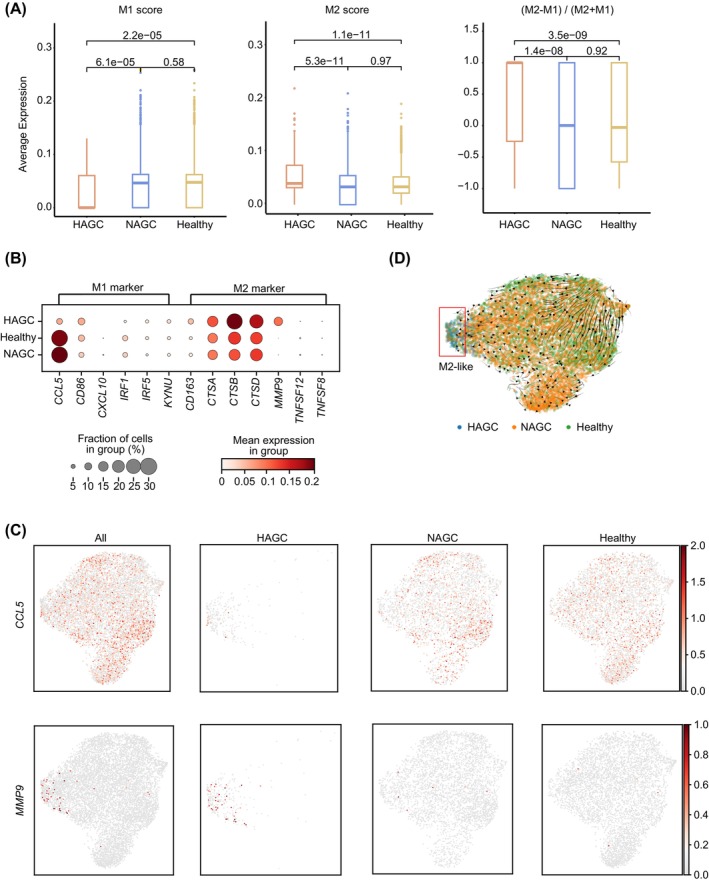
M2‐polarization characteristics of HAGC mononuclear phagocytes and reduced T cell function in HAGC patients. (A) Boxplot illustrating the M1 and M2 scores, calculated based on the gene sets listed in Table S2, for the MP fraction of cells across different conditions. Statistical significance was determined using a two‐sided unpaired Welch's *t*‐test. (B) Bubble plot displaying the expression level of selected M1/M2‐signature genes in myeloid cells from HAGC patients, NAGC patients, and healthy individuals. The color scheme represents the scaled mean expression in the group. The bubble size indicates the fraction of cells expressing marker genes under different conditions. (C) UMAP visualization demonstrating the expression patterns of a selected M1‐signature gene, *CCL5* (top), and an M2‐signature gene, *MMP9* (bottom), in each group. The color scheme represents the levels of gene expression. The UMAP plots are categorized based on the origin of disease or control. (D) scVelo RNA velocity analysis, projecting the velocity fields onto the UMAP distribution, to estimate macrophage polarization under different conditions.

Microphages, when activated, can polarize into two main subsets: classically activated M1 macrophages and alternatively activated M2 macrophages.^24^ M1 macrophages exhibit intrinsic phagocytic abilities and enhanced inflammatory response, thus behaving as anti‐tumour cells. On the other hand, M2 macrophages possess a diverse array of tumour‐promoting capabilities, including immunosuppression, angiogenesis and neovascularization. Intriguingly, analysis of M1‐ and M2‐specific gene expression revealed a significantly elevated M2 score and a decreased M1 score in HAGC MP cells, resulting in a biased overall M2‐like signature (Figure 3A). This finding was exemplified by the downregulation of the M1 marker gene *CCL5* and the expression of M2‐specific genes, such as *MMP9* and *CTSA/B/C* (Figure 3B, C). RNA velocity analysis further supported the transition of MPs towards an M2‐like state in HAGC (Figure 3D), suggesting the immunosuppressive properties of HAGC MPs.

### 2.4 Suppressed T cell activity in HAGC patients

**FIGURE 4:**
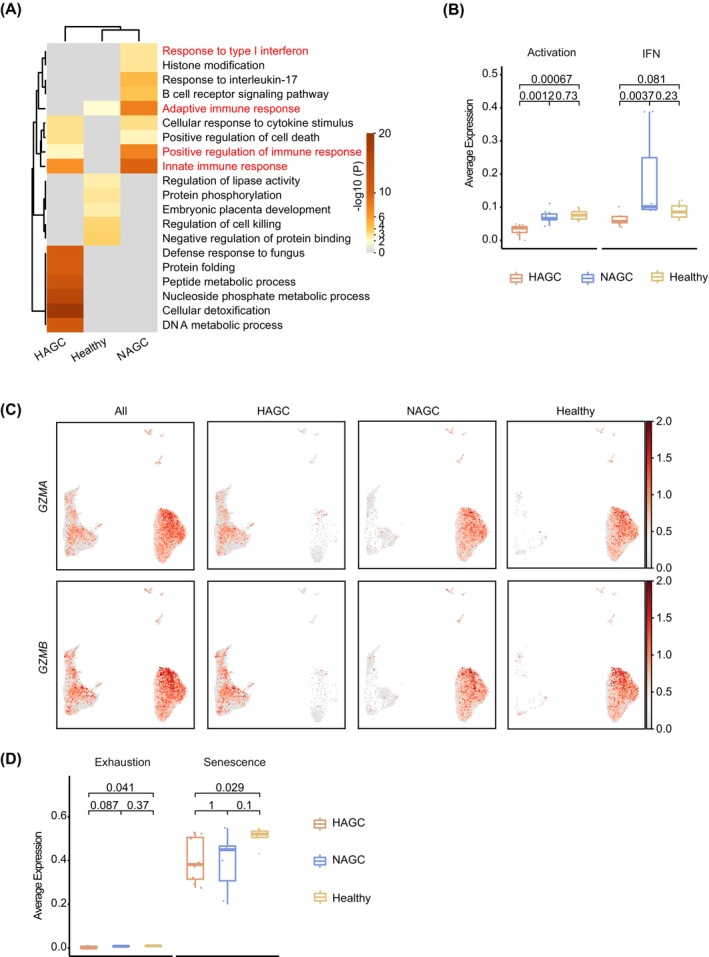
Reduced T cell function in HAGC patients. (A) Heatmap showing the enrichment of gene ontology terms associated with biological process in differentially expressed genes of T cells among the indicated groups. The color bar represents the –log10 transformed P value generated by Metascape. (B) Boxplots demonstrating the activation and IFN scores, calculated based on gene sets for T/NK cell populations, under different conditions. Statistical significance was determined using a two‐sided unpaired Welch's *t*‐test. (C) UMAP showing the expression of selected T cell‐activation genes *GZMA* (top) and *GZMB* (bottom) across the different conditions. The color scheme indicates the expression level. (D) Boxplots illustrating the exhaustion and senescence scores, calculated based on gene sets for T/NK cell populations, under different conditions. Statistical significance was calculated using a two‐sided unpaired Welch's *t*‐test.

Considering the immunosuppressive potential of MPs in patients with HAGC, we next investigated the responses of lymphoid cells. Compared to patients with NAGC and healthy individuals, B cell responses appeared relatively unaffected in HAGC patients (Figure 1E and Supplementary Figure S3A, Table S5). Gene ontology (GO) analysis of differentially expressed genes revealed that genes associated with type I interferon response, adaptive immune response, positive regulation of immune response and innate immune response were decreased in T cells of HAGC patients compared to NAGC patients (Figure 4A), suggesting the suppression of HAGC T cells. Indeed, the expression of genes associated with T cell activation and interferon was significantly lower in HAGC patients than in NAGC patients and healthy individuals (Figure 4B and Supplementary Figure S4A, B). T cell effector genes *GZMA* and *GZMB* exhibited low expression in HAGC patients too (Supplementary Figure S4C). These findings, combined with the reduced lymphocyte proportions in HAGC PBMCs (Figure 1E, F and Table S1), support the suppression of T cells in HAGC patients. However, further examination revealed that gene sets associated with cell exhaustion were expressed at a low level in HAGC, unlike tumour‐infiltrating T cells.^9^, ^10^, ^25^ Additionally, gene sets associated with senescence, including ribosome and histone genes, showed no significant differences between HAGC and NAGC, although there was a slight reduction compared to healthy controls (Figure 4D and Supplementary Figure S4C). Therefore, T cells in PBMCs of HAGC patients are suppressed but do not display signs of exhaustion or senescence.

### 2.5 HAGC myeloid cells suppress T cells

**FIGURE 5:**
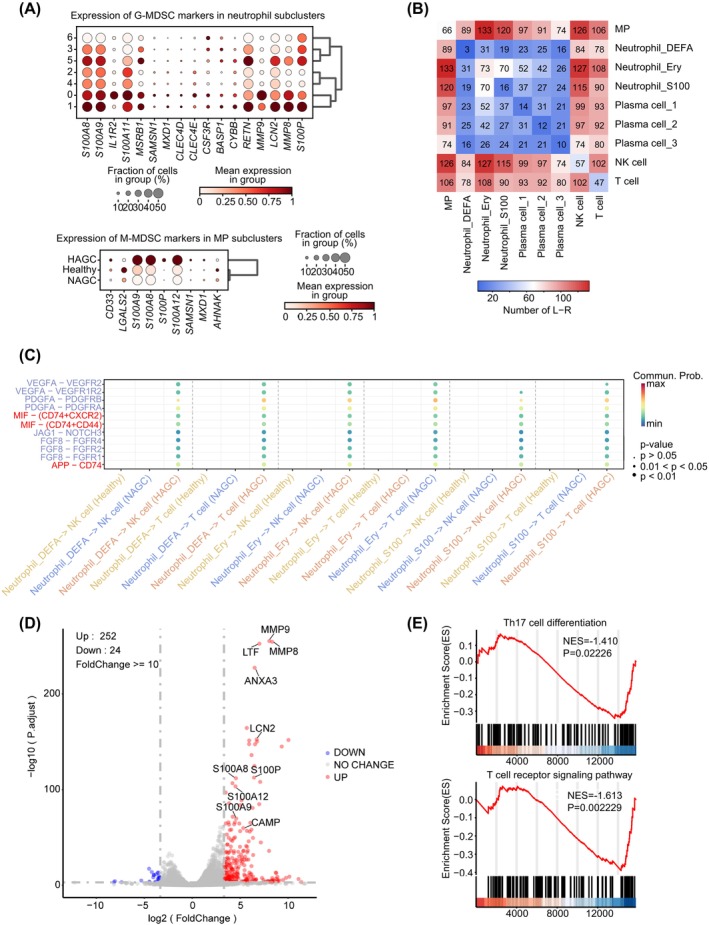
Suppressive crosstalk of myeloid cells on T cells. (A) Bubble plots showing the expression of G‐MDSC marker genes in neutrophil subclusters (top) and expression of M‐MDSC marker genes in MP clusters (bottom) across different conditions. The color scheme is based on the scaled mean expression in the respective group, ranging from 0 (white) to 1 (red). The bubble size indicates the fraction of cells expressing marker genes in each group. (B) Heatmap illustrating the count of interacting ligand‐receptor pairs between each cell type in HAGC cells, generated using CellChat. The color scheme represents the number of interacting receptor‐ligand pairs. (C) Bubble plot displaying ligand‐receptor pair putative interactions between neutrophils and T/NK cells, divided by diseased/healthy conditions. The color bar indicates the communication probability of each pair in each group. The size of the bubbles corresponds to the *P*‐value. (D) Volcano plot depicting the differentially expression of genes in PBMCs between HAGC and NAGC patients. The plot was generated based on fold change >10 and q‐value < 0.05 calculations obtained from bulk RNA‐seq. (E) Gene Set Enrichment Analysis (GSEA) results for differentially expressed genes between PBMCs of HAGC and NAGC patients. The analysis reveals a significant association of these genes with Th17 differentiation and T cell receptor signalling pathway.

Mononuclear phagocytes and granulocytes are crucial groups of innate immune cells that have essential roles in defending against pathogens. In disease settings, some of these cells display an immature morphology and exhibit immunosuppressive functions, which have been associated with negative prognostic outcomes and treatment responses. These cells are referred to as myeloid‐derived suppressor cells (MDSCs) and can be further categorized into granulocyte‐like MDSCs (G‐MDSC) and mononuclear phagocyte‐like MDSCs (M‐MDSCs).^26^, ^27^ The granulocyte and the mononuclear phagocyte are primarily composed of neutrophil and monocyte. Considering that HAGC PBMCs were primarily composed of active yet immature neutrophils, and MP cells exhibited an M2 immunosuppressive phenotype, we next investigated whether neutrophils and M2‐polarised MPs expressed G‐MDSC and M‐MDSC markers, respectively. Remarkably, neutrophil subsets 0, 1, and 5 of HAGC patients exhibited expression of G‐MDSC‐related markers (Figure 5A). M‐MDSC markers were also upregulated in M2‐like MPs in HAGC patients (Figure 5A). These findings suggest that myeloid cells in HAGC patients acquire the characteristics of MDSCs with immunosuppressive activity.

To elucidate the mechanism involved in T cell suppression, we explore the predicted crosstalk between different types of cells by analysing receptor‐ligand pairs. Correlation analysis indicated a close relationship among MPs, S100‐expressing neutrophils, Ery‐like neutrophils, T cells, and NK cells in HAGC patients (Figure 5B). Interestingly, we observed a higher number of predicted crosstalk interactions in HAGC patients compared to both NAGC patients and healthy controls (Supplementary Figure S5A). To further investigate cell‐cell communication between myeloid cells and T cells in HAGC patients, we utilized CellChat to identify potential ligand‐receptor pairs.^28^ Elevated levels of immunosuppressive signallings exerted from HAGC neutrophils to T cells and NK cells were observed, including APP‐CD74, MIF‐(CD74+CXCR2), and MIF‐(CD74+CD44) (Figure 5C and Supplementary Figure S5B).^29^ In addition, the signalling pathways of RETN‐CAP1 and ANXA1‐FPR1 from MPs to neutrophils, known to trigger cytokine storms,^30^ were significantly upregulated in HAGC patients, which is consistent with the hyper‐inflammatory status in HAGC (Supplementary Figure S5B–D).

To verify the reliability of scRNA‐seq results and gain further insights into HAGC, we conducted bulk RNA‐seq using PBMCs. When compared to PBMCs of NAGC, we identified 252 upregulated genes and 24 downregulated genes in HAGC PBMCs. Notably, markers associated with MDSC, including *MMP9*, *LCN2*, *S100A8*, *S00A9*, *S100A12* and *S100P* were significantly upregulated in HAGC PBMCs as compared to NAGC PBMCs (Figure 5D, Table S6). In addition, gene ontology (GO) analysis of the differentially expressed genes demonstrated a strong association of upregulated genes with the biological process of neutrophil‐mediated immunity. Enrichment of genes related to the response to foreign entities such as bacteria, fungi and toxic substances was observed in HAGC, suggesting the activation of innate immunity (Supplementary Figure S5E). Furthermore, gene set enrichment analysis (GSEA) indicated that genes involved in Th17 cell differentiation and T cell receptor signalling pathways were downregulated in HAGC compared to NAGC (Figure 5E), suggesting the repression of T cell function in HAGC.

Collectively, our findings support the notion that myeloid cell populations in HAGC patients effectively suppress T cell activity and induce systemic inflammatory responses. This validation through bulk RNA‐seq provides enhanced confidence in the scRNA‐seq results and facilitates a deeper understanding of the molecular characteristics of HAGC.

### 2.6 Aberrant cell‐cell communication and S100 family gene expression in neutrophils contributes to DIC development in HAGC

**FIGURE 6:**
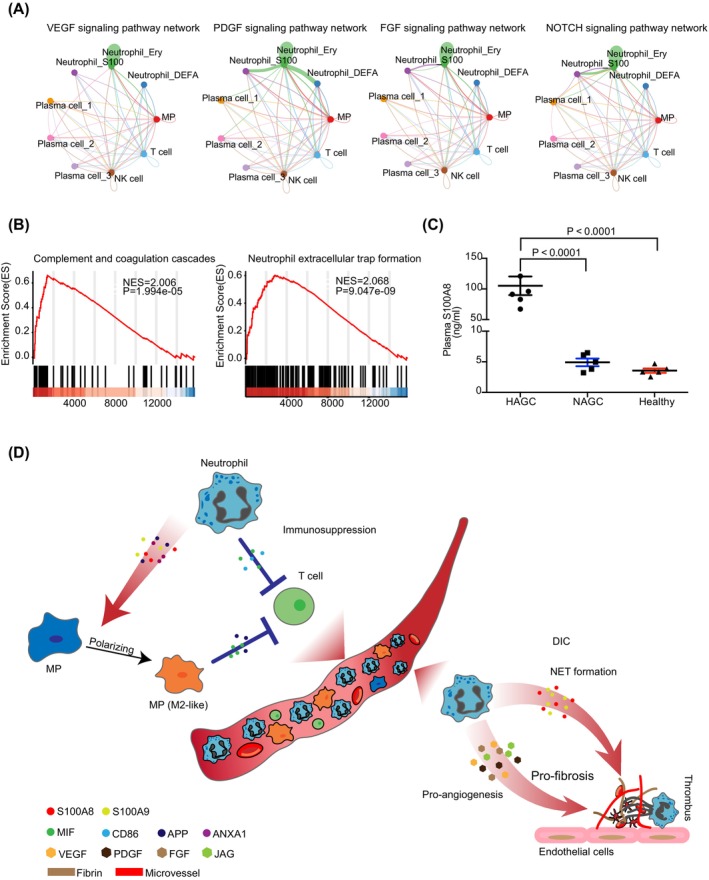
Enhanced fibrosis and angiogenesis signalling pathways and S100 expression in HAGC patients. (A) Circle plot showing the inferred VEGF/PDGF/FGF/NOTCH signalling pathway network strength in the HAGC group. (B) Gene set enrichment analysis (GSEA) results for differentially expressed genes between PBMCs of HAGC and NAGC patients. The analysis reveals a significant association of these genes with complement and coagulation cascades and neutrophil extracellular trap formation. (C) ELISA assay reveals that S100A8 protein is significantly upregulated in PBMCs of HAGC patients as compared to NAGC patients and healthy individuals. Statistical significance was determined using one‐way ANOVA. (D) Schematic model depicting the underlying mechanism by which HAGC contributes to the development of a complex systemic DIC coupled with immunosuppression of lymphoid cells.

Furthermore, we explored the potential mechanisms underlying DIC development in HAGC patients. Our analysis revealed a substantial activation of signalling pathways associated with angiogenesis and fibrosis, including VEGF, PDGF, FGF and NOTCH signalling pathways^31^, ^32^ (Figure 6A). Centrality analysis identified Neutrophil_Ery cells as the primary source of the ligands of these pathways (Supplementary Figure S6A), which may be involved in DIC pathogenesis by inducing the formation of microvessels and fibrous tissue (Supplementary Figure S6B).

Previous studies have implicated S100A8 and S100A9 in neutrophil NET formation, which has been further linked to thrombosis in DIC.^33^, ^34^ In our scRNA‐seq study of HAGC PBMCs, we observed a significant upregulation of S100A8 and S100A9 genes, particularly in neutrophils (Supplementary Figure S6C). This finding aligns with the observed DIC among HAGC patients. Additionally, our bulk RNA‐seq analysis comparing PBMCs from HAGC and NAGC patients confirmed the enhanced expression of S100A8 and S100A9 in HAGC (Figure 5D, Table S6). GSEA revealed an upregulation of genes associated with complement and coagulation cascades as well as NET formation, in HAGC patients (Figure 6B). To further validate these findings, we performed an ELISA assay using blood plasma and observed a significant increase in S100A8 protein levels in HAGC. This suggests that S100A8 may serve as a potential biomarker for HAGC diagnosis (Figure 6C).

## 3 DISCUSSION

In this study, we employed scRNA‐seq to examine the gene expression of HAGC PBMCs. In comparison with NAGC and healthy controls, we observed a substantial increase in neutrophil population in HAGC PBMCs, and these neutrophils were immature but active. MPs exhibited an M2‐like state. Together with MPs, neutrophils in HAGC patients suppress T/NK cell activity, at least partially by forming G‐MDSCs and M‐MDSCs. APP and MIF signalling pathways mediate this effect. Our data also shed light on the potential mechanisms underlying DIC development in HAGC. The activation of angiogenesis and fibrosis‐associated signalling pathways, along with the upregulation of S100A8 and S100A9 genes in neutrophils, provides insights into the pathogenesis of DIC in HAGC (Figure 6D).

It is worth noting that no neutrophil expansion was observed in previous scRNA‐seq studies of stomach tissues and PBMCs in non‐HAGC gastric cancer patients.^7^, ^8^, ^9^, ^10^ Neutrophil dysfunction has not previously been acknowledged as a characteristic of GC. Neutrophils play a crucial role as the first line of defense against infectious pathogens in the human body. However, clinical examination of HAGC patients revealed no signs of infection, suggesting that neutrophils may respond to other inflammatory or immunostimulatory signals resulting from HAGC. Notably, our study identified a significant increase in the ratio of immature neutrophils resembling G‐MDSCs in HAGC patients.^26^ These neutrophils suppressed T cell activity through multiple signalling pathways. This phenomenon could potentially be attributed to the extensive metastasis of tumour cells in the bone marrow which leads to complex systemic pro‐inflammatory myeloid cells coupled with the immunosuppression of lymphoid cells.

Moreover, DIC is a major pathological feature observed in HAGC patients, profoundly impacting their overall health.^2^ Previous studies unrelated to HAGC have demonstrated a correlation between DIC and higher levels of NET formation in patients with conditions such as COVID‐19 or sepsis.^35^, ^36^ Interestingly, genes vital for NET formation, particularly *S100A8* and *S100A9*, showed a substantial upregulation in HAGC patients. Recent studies have shown that *S100A8* and *S100A9* serve as markers of NETosis and can induce aberrant activation of neutrophils.^37^, ^38^ Strikingly, the blood of COVID‐19 patients with thrombosis contains high levels of circulating NETs, S100A8 and S100A9, along with an abundance of immature neutrophils in the bloodstream.^39^ Inhibition of S100A8/A9 by the inhibitor paquinimod has demonstrated effective amelioration of the immune disorder caused by COVID‐19.^38^ Considering the similar innate immune system abnormalities observed in HAGC and severe COVID‐19 patients, S100A8 and S100A9 may be promising diagnostic and therapeutic targets for DIC in HAGC patients.

In summary, our study provides important insights into the molecular and cellular mechanisms involved in HAGC patient immune response. We demonstrated the induction of activated but aberrant immature neutrophils in HAGC, which contribute to the immunosuppression of lymphoid cells and the activation of signalling pathways associated with DIC development. Our findings highlight the role of innate immunity in the pathogenesis of HAGC. Additionally, the identification of S100A8 and S100A9 as potential diagnostic and therapeutic targets further emphasizes their clinical significance in HAGC management.

## 4 METHODS

### 4.1 Collection of samples and clinical data

This study was approved by the Clinical Research Ethics Committee in the Sixth Affiliated Hospital, Sun Yat‐sen University (Number 2021ZSLYEC‐090). Informed consent was obtained from all patients before the study. The study included patients newly diagnosed with HAGC according to the diagnostic criteria for HAGC.^2^ NAGC is defined as unresectable, advanced, or recurrent GC according to the World Health Organization (WHO) criteria. HAGC was identified as gastric cancer characterized by BMM and DIC, with DIC being defined by the criteria established by the International Society on Thrombosis and Hemostasis (ISTH).^40^ Diffuse BMM was defined as instances that had a bone metastasis score ≥2, according to the extent of disease (EOD) criteria, on fluorodeoxdyglucose positron emission tomography/CT (FDG‐PET/CT) scans. Patients were excluded if they had any of the following conditions: hypertensive crisis, severe uncontrolled systemic comorbidities such as infection, severe cardiovascular disease, epilepsy, pregnancy or breastfeeding. Seven HAGC patients and six NAGC patients who satisfied the abovementioned inclusion and exclusion criteria were included in the scRNA‐seq study. The participants ranged in age from 20 to 80 years, with approximately equal male and female gender composition (Table S1). Clinical data were collected retrospectively from the medical records of patients diagnosed with HAGC or NAGC between 2019 and 2022, while haematological data were acquired from blood samples analysed through a standard haematology analyser to determine blood component levels.

### 4.2 Preparation of single‐cell suspension

PBMCs were isolated from fresh peripheral blood using Ficoll Hypaque Solution (Tianjin Haoyang Biological Manufacture Co., Ltd.) (https://www.tbdscience.com). The cells were then washed with 1× phosphate‐buffered saline (PBS) and treated with red blood cells lysis buffer (Beijing Solarbio Science & Technology Co., Ltd.) to remove red blood cells. Following the computation of cell count and viability, PBMCs were diluted to 10 × 10^4^ cells/mL using D‐PBS and subjected to microwell‐seq experiment.

### 4.3 Microwell‐seq

Sample preparation for scRNA‐seq was accomplished utilizing the microwell‐seq platform as described.^11^ Prior to the microwell‐seq experiment, a disposable agarose microwell plate was generated, as described previously. Magnetic beads (Suzhou Knowledge and Benefit Sphere Tech. Co., Ltd.) were conjugated with 1 × 10^8^ oligonucleotides through three rounds of split‐pooling. Cells and barcoded beads were pipetted into the microwell array for lysis. After reverse transcription, exonuclease I treatment, cDNA amplification, transposase fragmentation, and selective PCR, the samples were ready for sequencing for paired‐end 150 bp reads using the Illumina Hiseq X Ten system.

### 4.4 Bioinformatic analysis of microwell‐seq data

The FASTQ files of paired‐end sequencing were first aligned to hg38 genome assembly using STAR‐solo (2.7.10a),^41^ with the following parameters: ‘–soloType CB_UMI_Complex –soloCBposition 0_0_2_‐1 3_1_3_6 3_22_3_27 –soloFeatures Gene –soloUMIposition 3_28_3_33 –outFilterMultimapNmax 100 –winAnchorMultimapNmax 100 –outSAMmultNmax 1 –outSAMtype BAM SortedByCoordinate –twopassMode Basic’. BAM files generated were then filtered to exclude reads without valid barcodes, while reads and barcodes were quantified utilizing scTE.^42^ Subsequently, we used the Scanpy Python package (1.9.1)^43^ to merge the resulting quantification files into a single h5ad file, which was filtered by removing cells containing fewer than 300 genes or 700 counts and more than 5% of mitochondrial reads, as well as genes expressed in fewer than 50 cells (~0.1%). The resultant file was then normalized using the scran R package (1.20.1)^44^ and integrated with Python implementation of harmony.^45^ After analysing the data using Scanpy and obtaining highly variable genes, we clustered the resulting cells with the Leiden algorithm (Resolution = 0.9) and annotated the cell type using specific marker genes. To define the cluster marker, genes with a DE score above 3.5 are considered potential cluster markers. In addition, the genes with high DE scores, as well as those reported as cell cluster markers in previous single‐cell research, were given high priority and selected as cluster markers (Table S3). For the sub‐clustering of neutrophils, the annotated neutrophils were extracted and re‐clustered using the Leiden algorithm (Resolution = 0.8). Cell‐type‐specific genes with high DE scores were selected as markers for the sub‐clusters of neutrophils (Table S4).

### 4.5 Differentially expressed gene and gene ontology

The differentially expressed genes of each condition in different cell types were measured by ‘tl.rank_genes_groups’ of the Scanpy python package and the fold‐change >2 and adjusted‐*P* value <0.001 was set as the threshold. The differentially expressed genes in three conditions in each cell type were then subjected to Metascape^46^ to obtain enriched GO terms and heatmap.

### 4.6 Expression signature scores

The expression signature scores of PBMCs, like M1 score of MP cells, were calculated as average gene expression levels of the gene set for the signature. Briefly, to measure M1 polarization level of MP cells, the marker gene set including TNF‐alpha, IL‐12, iNOS and others was used to calculate M1 score (Table S2). First, the raw gene expression values of single‐cell RNA‐seq data were normalized to convert to a comparable scale. Next, the normalized expression values of the M1 marker genes for each cell in the dataset were summed to obtain the total M1 expression value of MPs. Afterward, the total M1 expression value was divided by the total number of M1 marker genes and by the total number of cells in MPs to calculate the average M1 score for each MP. A higher M1 score in a cell indicates a higher tendency towards an M1 phenotype. The list of marker gene sets for signatures studied in this manuscript is included in Table S2.

### 4.7 Plotting of microwell‐seq data

UMAP, dotplot, and violin plots were generated using the ‘pl.umap’, ‘pl.dotplot’ and ‘pl.violin’ functions from the Scanpy python package and respectively color‐coded based on their contents 43. Boxplots were generated using custom scripts to extract the proportions of cell types and the average expression of signature genes, with the ‘ggboxplot’ function of ggpubr R package (0.4.0) utilized for plotting. Custom scripts were used to extract and calculate the average expression levels of genes, and heatmaps were drawn using the pheatmap R package (1.0.12).

### 4.8 CytoTRACE analysis

The expression matrix of the measured cells was extracted from h5ad files with homemade scripts and subjected to the CytoTRACE R package (0.3.3) (https://cytotrace.stanford.edu).^17^ Pseudotime was estimated using the ‘CytoTRACE’ function with default settings and the plots were drawn using the ‘plotCytoTRACE’ function with default settings. The results were combined with cell phenotype and UMAP information to produce the final plots.

### 4.9 scVelo analysis

The FASTQ files were aligned to the hg38 assembly using STAR‐solo with the scVelo parameters ‘–soloCellFilter None –soloFeatures Gene Velocyto’.^41^ The STAR‐solo results and scTE expression matrices were merged using custom scripts (https://github.com/oaxiom/sc_utils) and then packed into a combined file. Next, annotated neutrophils or MP cells were extracted from the complete dataset to generate an RNA dynamical velocity plot utilizing the scVelo Python package (0.2.5) (https://scvelo.readthedocs.io).^18^

### 4.10 Cell type crosstalk analysis

CellChat was used to quantify the intercellular communication networks and predict the main signal pathways based on scRNA‐seq data and investigate the differences in cell‐cell crosstalk among different cell types.^28^ Briefly, a CellChat object was constructed using the H5Seurat files derived from the Scanpy file. The major inputs and outputs of signalling were evaluated for all cell clusters with the aid of CellChatDB.human. The netVisual circle was employed to depict the number and strength of interactions among all cell clusters in each group. The netVisual bubble and netVisual circle were utilized to display significant ligand‐receptor interactions between the target cell clusters. Finally, netAnalysis_compute Centrality functions were used to select critical signalling pathways and investigate their influence on each cell cluster.

### 4.11 RNA‐seq

Whole RNA of PBMCs were purified using TRIzol reagent. The transcriptome library was generated by BGI and sequencing was performed on the BGISEQ‐500 platform. Raw data were aligned to human hg19 genome by HISAT2. The differentially expressed genes were identified by the ‘DESeq2’ R package. GO and GSEA analyses were conducted using the ‘clusterProfiler’ R package.

### 4.12 ELISA

S100A8 protein levels in the plasma samples were measured using a commercially available enzyme‐linked immunosorbent assay (ELISA) kit (Wuhan Fine Biotech Co., Ltd). The assay was performed following the manufacturer's instructions and protocol.

### 4.13 Statistical analysis

A minimum of five samples for each condition were included in the analysis to ensure robust and comprehensive results. Statistical analysis was performed using two‐sided unpaired Mann‐Whitney U test, two‐sided unpaired Welch's *t*‐test, or one‐way ANOVA to determine significant differences when two conditions or three conditions were compared. *P*‐value less than 0.05 indicates statistical significance in the observed differences.

## AUTHOR CONTRIBUTIONS

Ping Yuan and Jian Xiao initiated the study; Rui Ma, Chuyue Wang, Rong Hu, You Chen, Yingying Zhao and Weili Wu conducted the experiments; Xuemeng Zhao, Liyang Shi and Jiao Chen performed bioinformatics analysis. Xiaohui Zhai, Taiyuan Cao, Ge Du and Shanshan Li collected samples. Xing Fang, Yuan Liao, Mengmeng Jiang, Junqing Wu, Renying Wang, Haide Chen and Guoji Guo advised the experimental techniques. Lifeng Ma and Qizhou Lian performed data analysis. Andrew P. Hutchins and Ping Yuan supervised the study. Rui Ma, Xuemeng Zhou, Andrew P. Hutchins and Ping Y wrote the manuscript. All authors have read and approved the final manuscript.

## FUNDING INFORMATION

The key project funding in the Sixth Affiliated Hospital, Sun Yat‐sen University (2022JBGS13 to P.Y.). [Correction added on 13 March 2024, after first online publication: funding for author Ping Yuan has been added].

## CONFLICT OF INTEREST STATEMENT

The authors declare no conflicts of interest.

## PATIENT CONSENT STATEMENT

Informed consent was obtained from all patients before the study.

## Supporting information


**Supplementary Figure S1.** Single cell RNA‐seq data analysis of PBMCs in HAGC patients, NAGC patients and healthy controls.
(A) Violin plots illustrating the average number of genes detected per patient sample (left) and the average number of counts per cell for each patient sample (right). ‘HP’ represents HAGC patients, ‘NP’ represents NAGC patients and ‘P’ represents healthy donors. (B) Pie chart displaying the distribution of cells within each group among all the successfully filtered cells. (C) UMAP visualization depicting the expression pattern of canonical marker genes within each cluster, with color‐coded representation based on the expression of selected marker genes. The colour scheme indicates the expression level of the respective genes. Erythroblasts (HBB, HBA1), neutrophils (DEFA3, DEFA1, S100A9, S100A8), monocytes (LYZ, CD14), T cells (CD3D, CD3E), NK cells (CCL5 and NKG7), and B cells (JCHAIN, IGHA1, IGLC2, IGLL5). (D) Scaled bar chart showing the proportion of cell types in each sample.
**Supplementary Figure S2.** Characteristics of neutrophils in HAGC. (A) Split UMAP visualization displaying the distribution of three clusters (Neutrophil_DEFA, Neutrophil_Ery, and Neutrophil_S100) within neutrophils, colour coded by the origin of each cluster. (B) UMAP representation of the expression patterns of canonical marker genes within each subcluster (Figure 2A), with color‐coding based on the expression of selected genes. The colour scheme reflects the expression level of each gene. Group 0 (HBA2, HBB), Group 1 (LTF, CAMP), Group 2 (IFI30, AC007192.1), Group 3/5 (DEFA1, DEFA1B, DEFA3), Group 4 (L1PA6, TMSB10), and Group 6 (ELANE, PRTN3, MPO). (C) Boxplot demonstrating the predicted differentiation score of neutrophil subclusters in HAGC, NAGC, and healthy individuals by CytoTRACE. (D) scVelo RNA velocity estimating the interrelationship between neutrophil subclusters. The velocity fields were projected onto the UMAP distribution. (E) UMAP representation of neutrophils, showing their pseudotime based on published BMMC of healthy individuals (highlighted in red) and HAGC PBMCs in this study. Cells are coloured according to CytoTRACE‐predicted ordering from 1 (red for less 2 differentiated) to 0 (blue for more differentiated) in the left plot and by cell type in the right plot. (F) Boxplot illustrating the predicted differentiation score obtained through CytoTRACE for published bone marrow neutrophil (highlighted in red) and PBMC neutrophil subclusters from this study. (G) Bubble plots depicting the expression patterns of primary, secondary, tertiary granule, and maturation genes in the neutrophil subclusters in the HAGC group. The colour scheme is based on the mean expression in the group, ranging from 0 (white) to 3 (red). The bubble size corresponds to the fraction of cells expressing the marker genes in the respective group. (H) Heatmap illustrating the expression patters of activation genes in neutrophils across different samples. The colour bar indicates the normalized expression level of each gene relative to all samples. The abbreviations ‘HP’ represent HAGC patients, ‘NP’ represent NAGC patients, and ‘P’ represent healthy donors.
**Supplementary Figure S3.** Unchanged B cell responses in HAGC. (A) Boxplot demonstrating the distributions of B cell populations in HAGC, NAGC, and healthy control samples. The statistical significance was calculated using a two‐sided unpaired Welch's *t*‐test.
**Supplementary Figure S4.** Suppressed T cell activity in HAGC. (A) Heatmap illustrating the expression patterns of activated genes in NK/T cells across different samples. The colour bar indicates the normalized expression level of each gene relative to all samples. The abbreviations ‘HP’ refers represent HAGC patients, ‘NP’ represent NAGC patients, and ‘P’ represent healthy donors. (B) Heatmap displaying the expression of IFN genes in NK/T cells among the indicated samples. The colour bar represents the normalized expression level of each gene relative to all samples. The abbreviations ‘HP’ represent HAGC patients, ‘NP’ represent NAGC patients, and ‘P’ represent healthy donors. (C) Heatmap illustrating the expression patterns of senescence‐related genes (histone and ribosomal genes) in NK/T cells among the indicated samples. The colour bar indicates the normalized expression level of each gene relative to all samples. The abbreviations 3 ‘HP’ represent HAGC patients, ‘NP’ represent NAGC patients, and ‘P’ represent healthy donors.
**Supplementary Figure S5.** Dysregulated signalling pathways in myeloid and lymphoid cells in PBMCs in HAGC. (A) Circle plots showing the numbers of interacted ligand‐receptor pairs between each two immune cell types in HAGC (left), NAGC (middle), and healthy control (left). (B) UMAP demonstrating the expression patterns of APP, MIF, ANXA1 and RETN across all cells and in HAGC, NAGC, and healthy groups. The colour scheme indicates the expression level of each gene. (C) Bubble diagram illustrating the ligand‐receptor pair interactions between neutrophils and MP, divided by diseased and healthy conditions. The colour bar indicates the communication probability of each pair in each group. The size of the bubbles represents the *P*‐value. (D) Bubble diagram depicting the ligand‐receptor pair interactions between MP and neutrophils, divided by diseased and healthy conditions. The colour bar indicates the communication probability of each pair in each group. The size of the bubbles represents the *P*‐value. (E) Gene ontology (GO) of upregulated genes in PMBCs between HAGC and NAGC patients.
**Supplementary Figure S6.** Signalling pathway associated with DIC in HAGC. (A) Heatmap presenting the role and importance of each cluster within the VEGF/PDGF/FGF/NOTCH signalling pathway network. The colour bar represents the importance of each component of the pathway network in the specific cell types, as quantified by Cellchat. (B) Schematic model showcasing the involvement of neutrophils in the HAGC group and potential signalling pathways implicated in DIC. These pathways activate signalling pathways associated with fibrosis and angiogenesis. (C) UMAP showing the expression of S100A8 (top) and S100A9 (bottom) under different conditions. The colour scheme indicates expression levels.

**Table S1.** Sample information and clinical blood test result.

**Table S2.** Lists of marker genes used to score studied features.

**Table S3.** Marker gene information for each cluster of PBMCs from all samples in single‐cell RNA‐sequencing dataset.

**Table S4.** Marker gene information for each subcluster of neutrophils from all samples in the single‐cell RNA‐sequencing dataset.

**Table S5.** Differentially expressed genes of T cells among HAGC patients, NAGC patients and healthy individuals in single‐cell RNAsequencing dataset.

**Table S6.** Differentially expressed genes between HAGC PBMCs and NAGC PBMCs in bulk RNA sequencing dataset.

## Data Availability

The data that support the findings of this study are available from the corresponding author upon reasonable request. scRNA‐seq raw data and RNA‐seq raw data reported in this paper have been deposited in Genome Sequence Archive (GSA) with the accession number HRA003949.
