# Supplementary material for: Single‐cell RNA sequencing reveals immune cell dysfunction in the peripheral blood of patients with highly aggressive gastric cancer
Source: Cell Prolif. 2024 Feb 6;57(5):e13591. doi: 10.1111/cpr.13591 (PMC11056698; doi:10.1111/cpr.13591)

Supplementary Figures

Supplementary Figure S1. Single cell RNA-seq data analysis of PBMCs in HAGC patients, NAGC patients and healthy controls.

(A) Violin plots illustrating the average number of genes detected per patient sample (left) and the average number of counts per cell for each patient sample (right). "HP" represents HAGC patients, "NP" represents NAGC patients, and "P" represents healthy donors.

(B) Pie chart displaying the distribution of cells within each group among all the successfully filtered cells.

(C) UMAP visualization depicting the expression pattern of canonical marker genes within each cluster, with color-coded representation based on the expression of selected marker genes. The color scheme indicates the expression level of the respective genes. Erythroblasts (*HBB*, *HBA1*), neutrophils (*DEFA3*, *DEFA1*, *S100A9*, *S100A8*), monocytes (*LYZ*, *CD14*), T cells (*CD3D*, *CD3E*), NK cells (*CCL5* and *NKG7*), and B cells (*JCHAIN*, *IGHA1*, *IGLC2*, *IGLL5*).

(D) Scaled bar chart showing the proportion of cell types in each sample.

Supplementary Figure S2. Characteristics of neutrophils in HAGC

(A) Split UMAP visualization displaying the distribution of three clusters (Neutrophil_DEFA, Neutrophil_Ery, and Neutrophil_S100) within neutrophils, color-coded by the origin of each cluster.

(B) UMAP representation of the expression patterns of canonical marker genes within each subcluster (**Figure 2A**), with color-coding based on the expression of selected genes. The color scheme reflects the expression level of each gene. Group 0 (*HBA2*, *HBB*), Group 1 (*LTF*, *CAMP*), Group 2 (*IFI30*, *AC007192.1*), Group 3/5 (*DEFA1*, *DEFA1B*, *DEFA3*), Group 4 (*L1PA6*, *TMSB10*), and Group 6 (*ELANE*, *PRTN3*, *MPO*).

(C) Boxplot demonstrating the predicted differentiation score of neutrophil subclusters in HAGC, NAGC, and healthy individuals by CytoTRACE.

(D) scVelo RNA velocity estimating the interrelationship between neutrophil subclusters. The velocity fields were projected onto the UMAP distribution.

(E) UMAP representation of neutrophils, showing their pseudotime based on published BMMC of healthy individuals (highlighted in red) and HAGC PBMCs in this study. Cells are colored according to CytoTRACE-predicted ordering from 1 (red for less

differentiated) to 0 (blue for more differentiated) in the left plot and by cell type in the right plot.

(F) Boxplot illustrating the predicted differentiation score obtained through CytoTRACE for published bone marrow neutrophil (highlighted in red) and PBMC neutrophil subclusters from this study.

(G) Bubble plots depicting the expression patterns of primary, secondary, tertiary granule, and maturation genes in the neutrophil subclusters in the HAGC group. The color scheme is based on the mean expression in the group, ranging from 0 (white) to 3 (red). The bubble size corresponds to the fraction of cells expressing the marker genes in the respective group.

(H) Heatmap illustrating the expression patters of activation genes in neutrophils across different samples. The color bar indicates the normalized expression level of each gene relative to all samples. The abbreviations “HP” represent HAGC patients, “NP” represent NAGC patients, and “P” represent healthy donors.

Supplementary Figure S3. Unchanged B cell responses in HAGC

(A) Boxplot demonstrating the distributions of B cell populations in HAGC, NAGC, and healthy control samples. The statistical significance was calculated using a two-sided unpaired Welch’s t-test.

Supplementary Figure S4. Suppressed T cell activity in HAGC

(A) Heatmap illustrating the expression patterns of activated genes in NK/T cells across different samples. The color bar indicates the normalized expression level of each gene relative to all samples. The abbreviations “HP” refers represent HAGC patients, “NP” represent NAGC patients, and “P” represent healthy donors.

(B) Heatmap displaying the expression of IFN genes in NK/T cells among the indicated samples. The color bar represents the normalized expression level of each gene relative to all samples. The abbreviations “HP” represent HAGC patients, “NP” represent NAGC patients, and “P” represent healthy donors.

(C) Heatmap illustrating the expression patterns of senescence-related genes (histone and ribosomal genes) in NK/T cells among the indicated samples. The color bar indicates the normalized expression level of each gene relative to all samples. The abbreviations

"HP" represent HAGC patients, "NP" represent NAGC patients, and "P" represent healthy donors.

Supplementary Figure S5. Dysregulated signaling pathways in myeloid and lymphoid cells in PBMCs in HAGC

(A) Circle plots showing the numbers of interacted ligand-receptor pairs between each two immune cell types in HAGC (left), NAGC (middle), and healthy control (left).

(B) UMAP demonstrating the expression patterns of *APP, MIF, ANXA1* and *RETN* across all cells and in HAGC, NAGC, and healthy groups. The color scheme indicates the expression level of each gene.

(C) Bubble diagram illustrating the ligand-receptor pair interactions between neutrophils and MP, divided by diseased and healthy conditions. The color bar indicates the communication probability of each pair in each group. The size of the bubbles represents the P-value.

(D) Bubble diagram depicting the ligand-receptor pair interactions between MP and neutrophils, divided by diseased and healthy conditions. The color bar indicates the communication probability of each pair in each group. The size of the bubbles represents the P-value.

(E) Gene ontology (GO) of upregulated genes in PMBCs between HAGC and NAGC patients.

Supplementary Figure S6. Signaling pathway associated with DIC in HAGC

(A) Heatmap presenting the role and importance of each cluster within the VEGF/PDGF/FGF/NOTCH signaling pathway network. The color bar represents the importance of each component of the pathway network in the specific cell types, as quantified by Cellchat.

(B) Schematic model showcasing the involvement of neutrophils in the HAGC group and potential signaling pathways implicated in DIC. These pathways activate signaling pathways associated with fibrosis and angiogenesis.

(C) UMAP showing the expression of *S100A8* (top) and *S100A9* (bottom) under different conditions. The color scheme indicates expression levels.

Figure S1

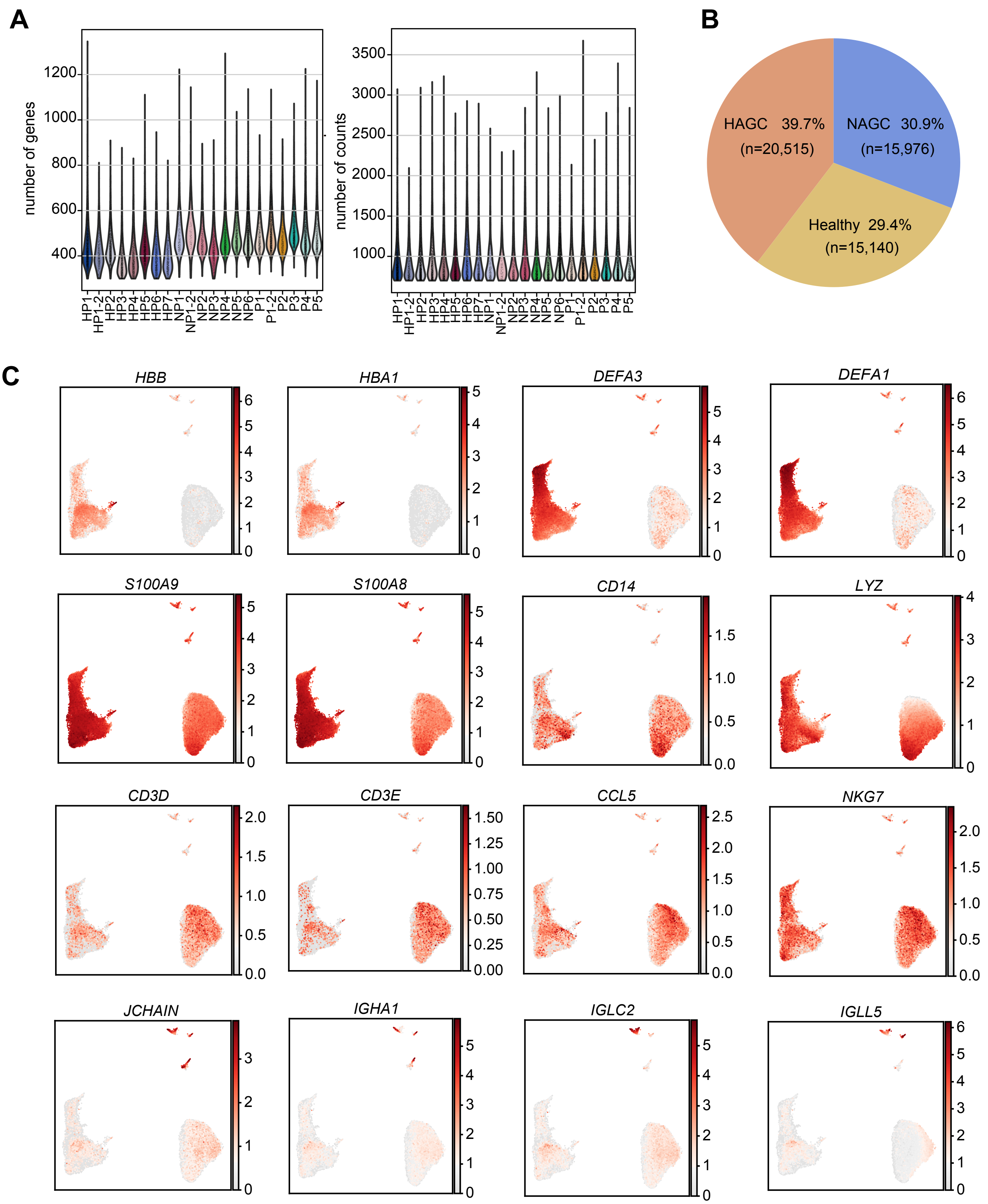
A
number of genes
number of counts
HP1
HP1-2
HP2
HP3
HP4
HP5
HP6
HP7
NP1
NP1-2
NP2
NP3
NP4
NP5
NP6
P1
P1-2
P2
P3
P4
P5
B
HAGC 39.7%
(n=20,515)
NAGC 30.9%
(n=15,976)
Healthy 29.4%
(n=15,140)
C
HBB
HBA1
DEFA3
DEFA1
S100A9
S100A8
CD14
LYZ
CD3D
CD3E
CCL5
NKG7
JCHAIN
IGHA1
IGLC2
IGLL5

Figure S1

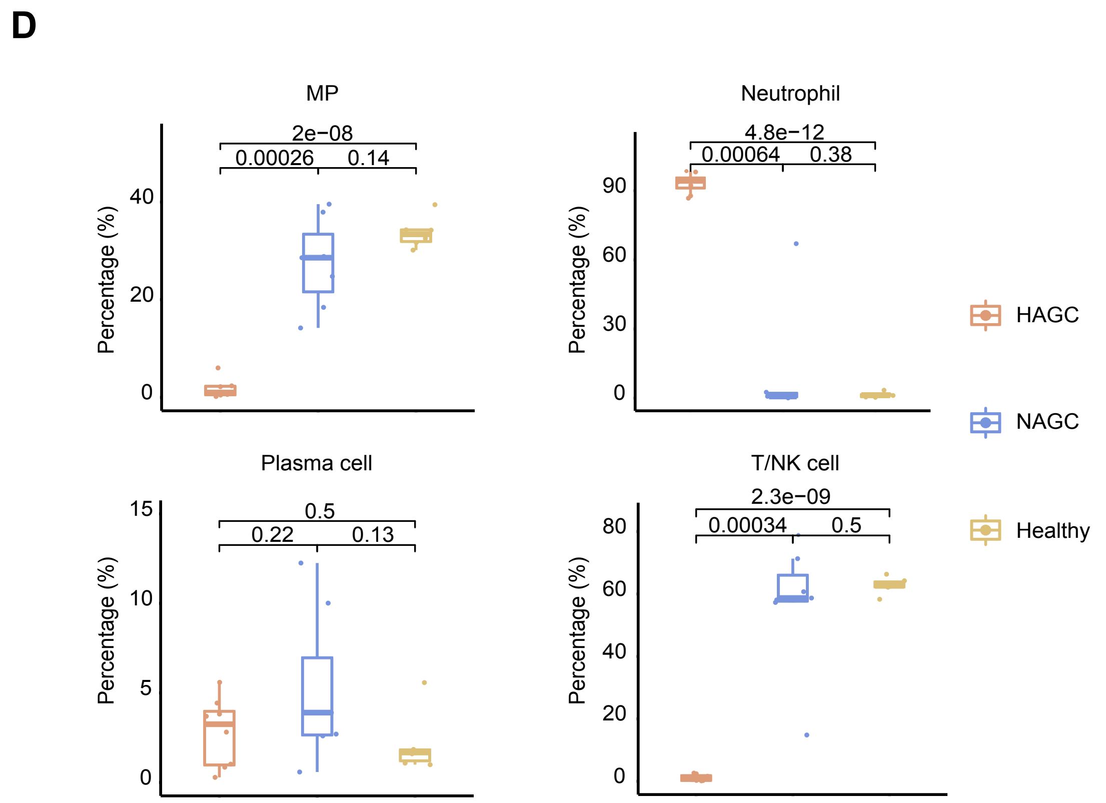
D
MP
2e−08
0.00026
0.14
Percentage (%)
40
20
0
Neutrophil
4.8e−12
0.00064
0.38
90
60
30
0
HAGC
NAGC
Healthy
Plasma cell
0.5
0.22
0.13
15
10
5
0
T/NK cell
2.3e−09
0.00034
0.5
80
60
40
20
0

Figure S2

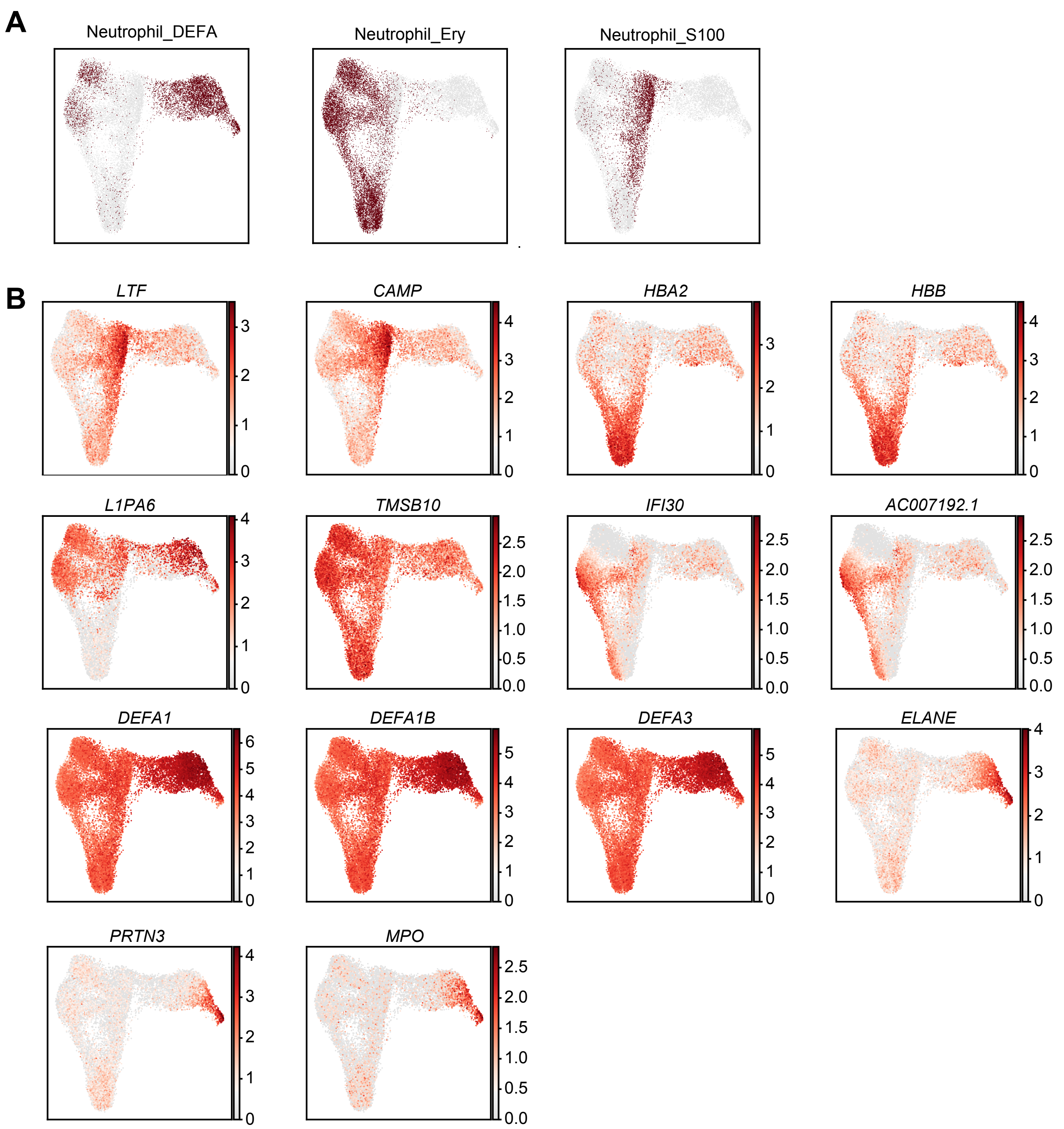
A
Neutrophil_DEFA
Neutrophil_Ery
Neutrophil_S100
B
LTF
CAMP
HBA2
HBB
L1PA6
TMSB10
IFI30
AC007192.1
DEFA1
DEFA1B
DEFA3
ELANE
PRTN3
MPO

Figure S2

C

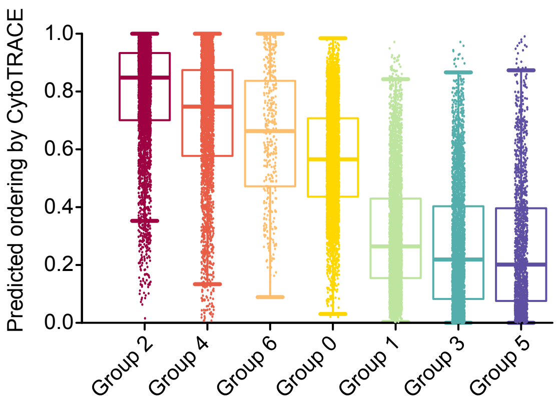

D

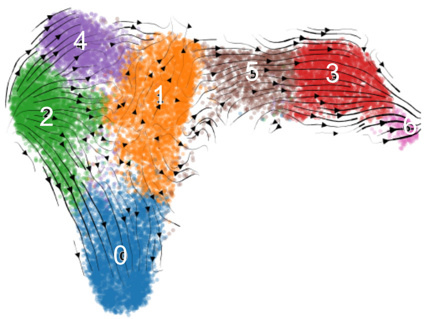

E

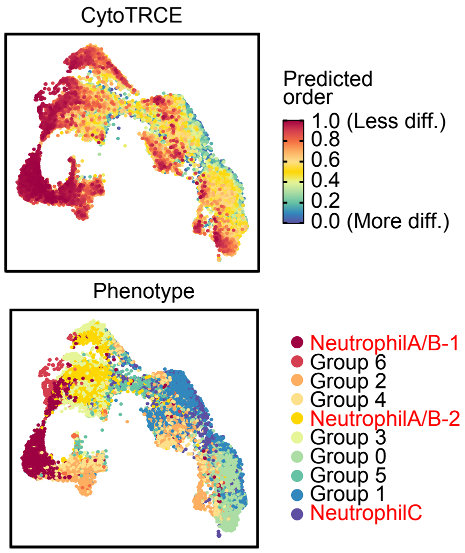

F

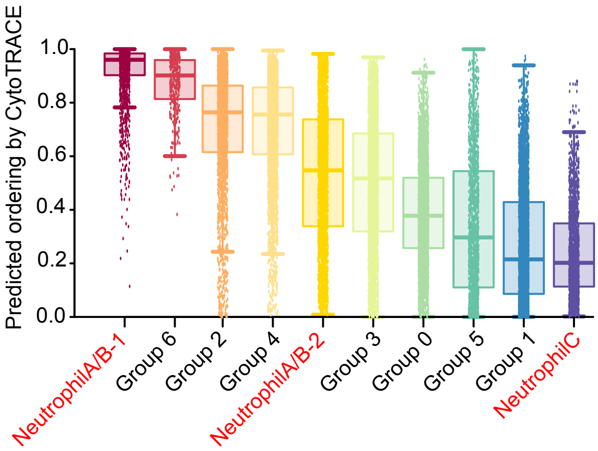

Figure S2

G

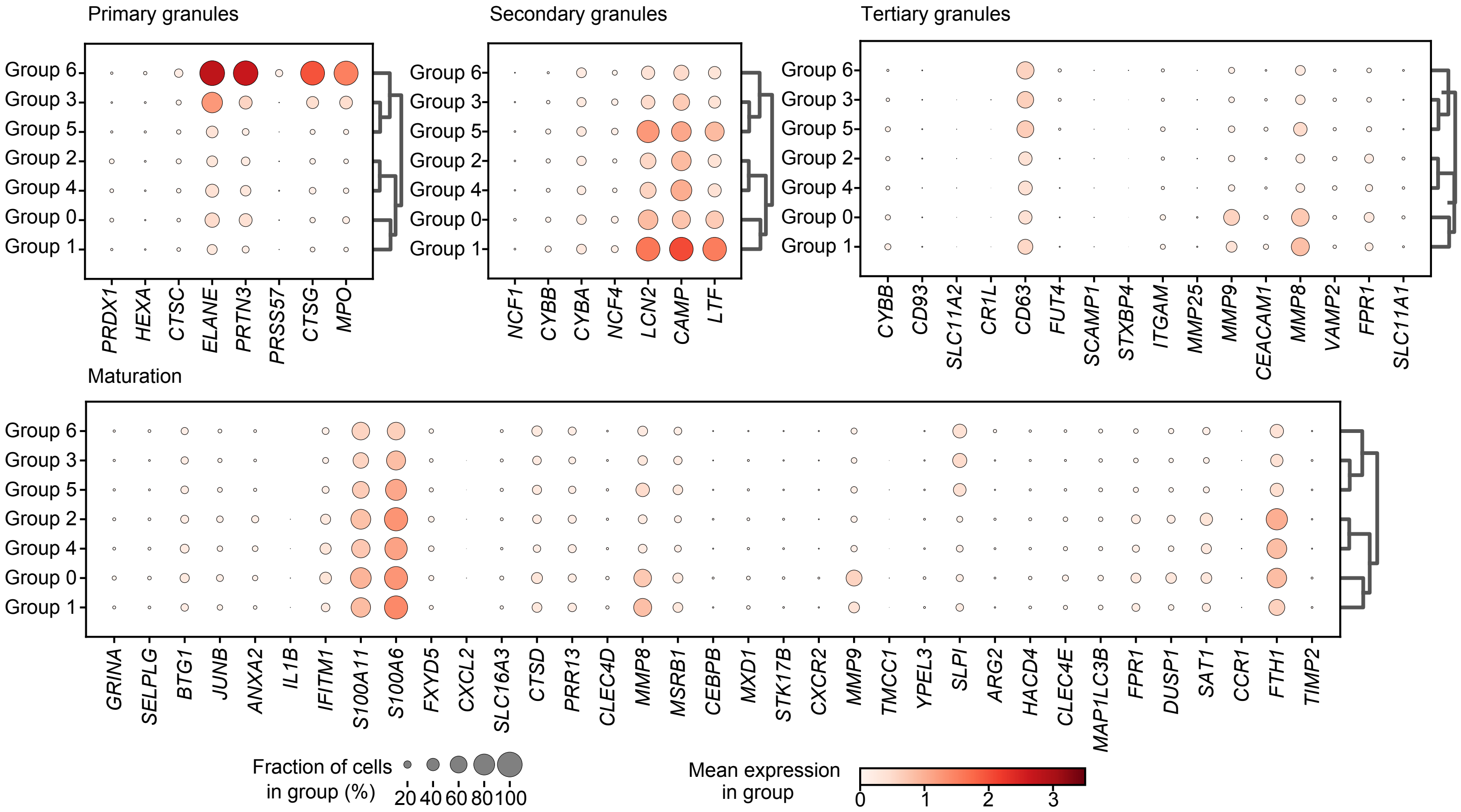
Primary granules
Secondary granules
Tertiary granules
Maturation
Group 6
Group 3
Group 5
Group 2
Group 4
Group 0
Group 1
PRDX1 HEXA CTSC ELANE PRTN3 PRSS57 CTSG MPO
NCF1 CYBB CYBA NCF4 LCN2 CAMP LTF
CYBB CD93 SLC11A2 CR1L CD63 FUT4 SCAMP1 STXBP4 ITGAM MMP25 MMP9 CEACAM1 MMP8 VAMP2 FPR1 SLC11A1
GRINA SELPLG BTG1 JUNB ANXA2 IL1B IFITM1 S100A11 S100A6 FXYD5 CXCL2 SLC16A3 CTSD PRR13 CLEC4D MMP8 MSRB1 CEBPB MXD1 STK17B CXCR2 MMP9 TMCC1 YPEL3 SLPI ARG2 HACD4 CLEC4E MAP1LC3B FPR1 DUSP1 SAT1 CCR1 FTH1 TIMP2
Fraction of cells in group (%)
20 40 60 80 100
Mean expression in group
0 1 2 3

H

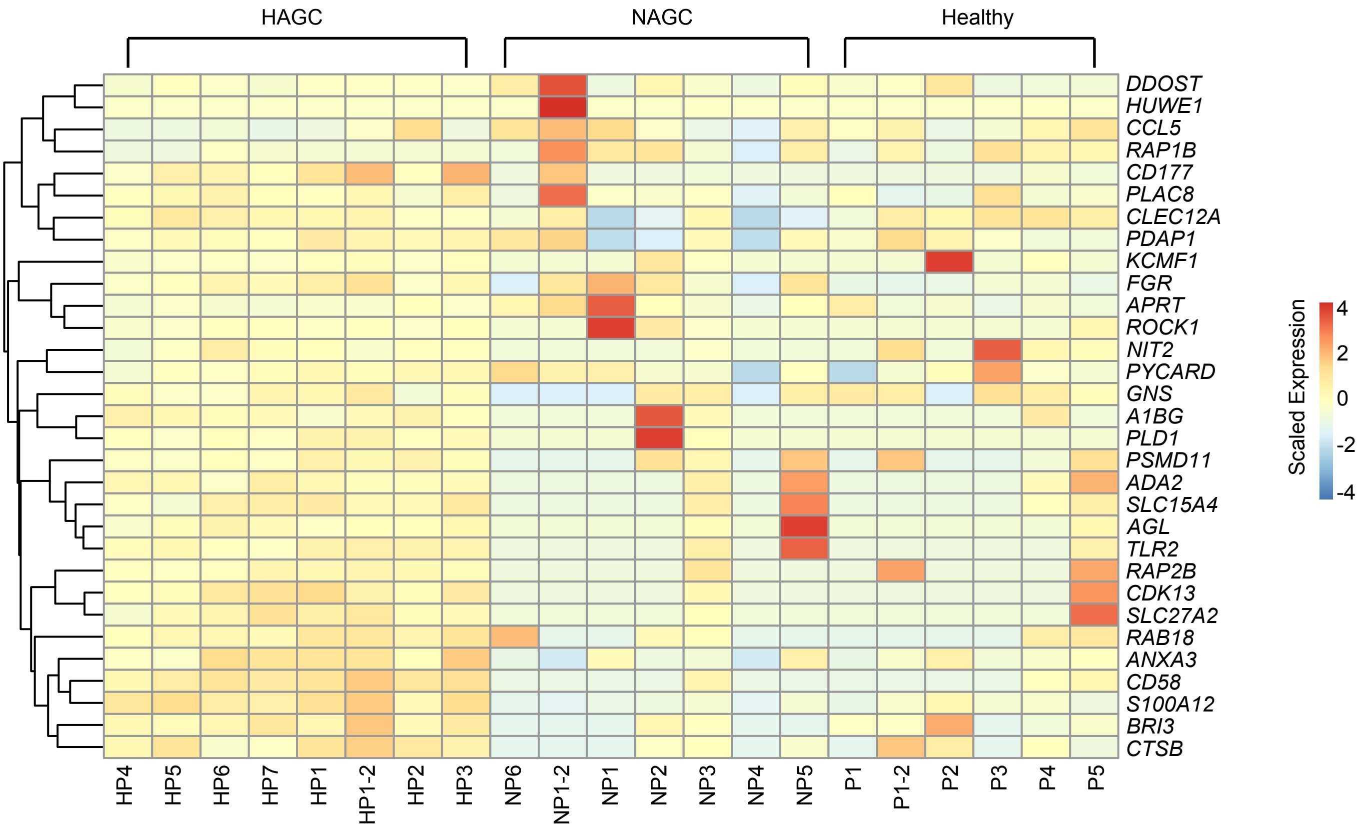
HAGC
NAGC
Healthy
DDOST
HUWE1
CCL5
RAP1B
CD177
PLAC8
CLEC12A
PDAP1
KCMF1
FGR
APRT
ROCK1
NIT2
PYCARD
GNS
A1BG
PLD1
PSMD11
ADA2
SLC15A4
AGL
TLR2
RAP2B
CDK13
SLC27A2
RAB18
ANXA3
CD58
S100A12
BRI3
CTSB
HP4 HP5 HP6 HP7 HP1 HP1-2 HP2 HP3 NP6 NP1-2 NP1 NP2 NP3 NP4 NP5 P1 P1-2 P2 P3 P4 P5
Scaled Expression
4
2
0
-2
-4

Figure S3

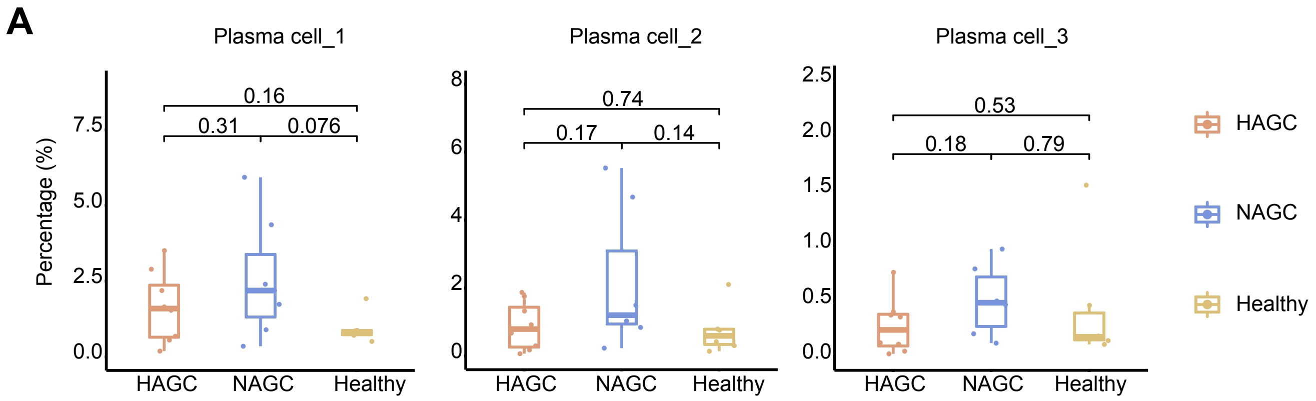
A
Plasma cell_1
Plasma cell_2
Plasma cell_3
Percentage (%)
7.5
5.0
2.5
0.0
0.16
0.31
0.076
HAGC
NAGC
Healthy
8
6
4
2
0
0.74
0.17
0.14
2.5
2.0
1.5
1.0
0.5
0.0
0.53
0.18
0.79
HAGC
NAGC
Healthy

Figure S4

A

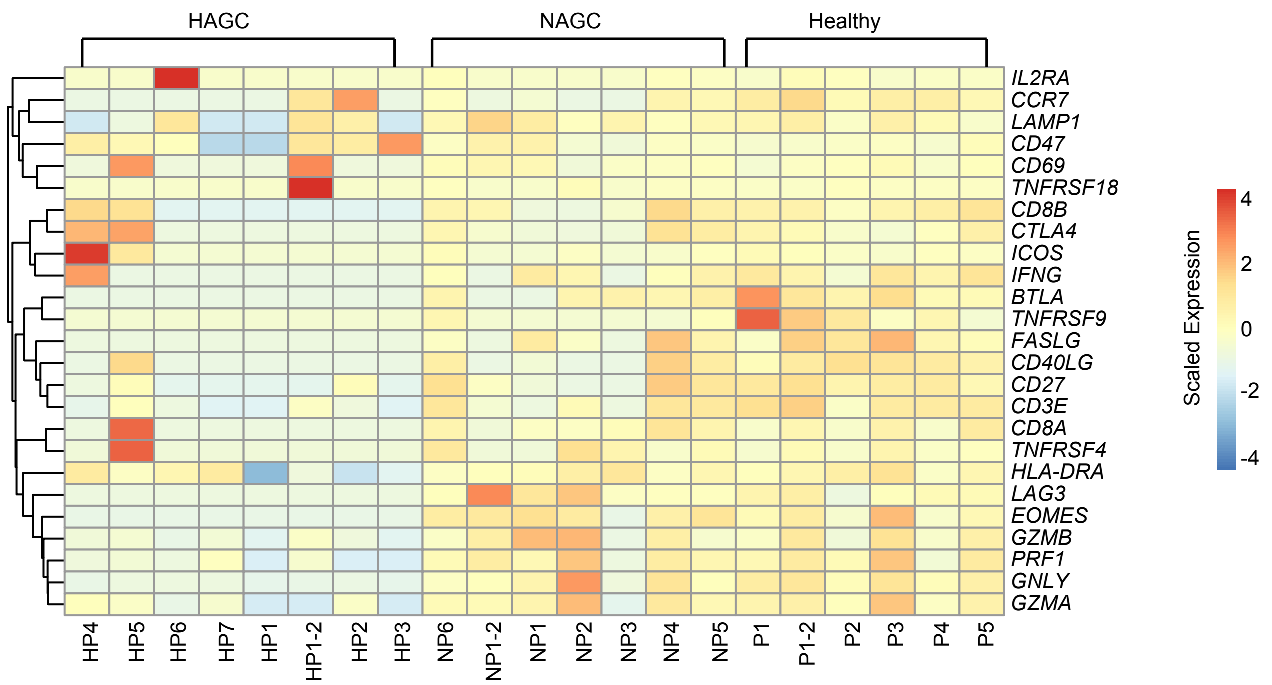
HAGC
NAGC
Healthy
IL2RA
CCR7
LAMP1
CD47
CD69
TNFRSF18
CD8B
CTLA4
ICOS
IFNG
BTLA
TNFRSF9
FASLG
CD40LG
CD27
CD3E
CD8A
TNFRSF4
HLA-DRA
LAG3
EOMES
GZMB
PRF1
GNLY
GZMA
HP4
HP5
HP6
HP7
HP1
HP1-2
HP2
HP3
NP6
NP1-2
NP1
NP2
NP3
NP4
NP5
P1
P1-2
P2
P3
P4
P5
Scaled Expression
4
2
0
-2
-4

B

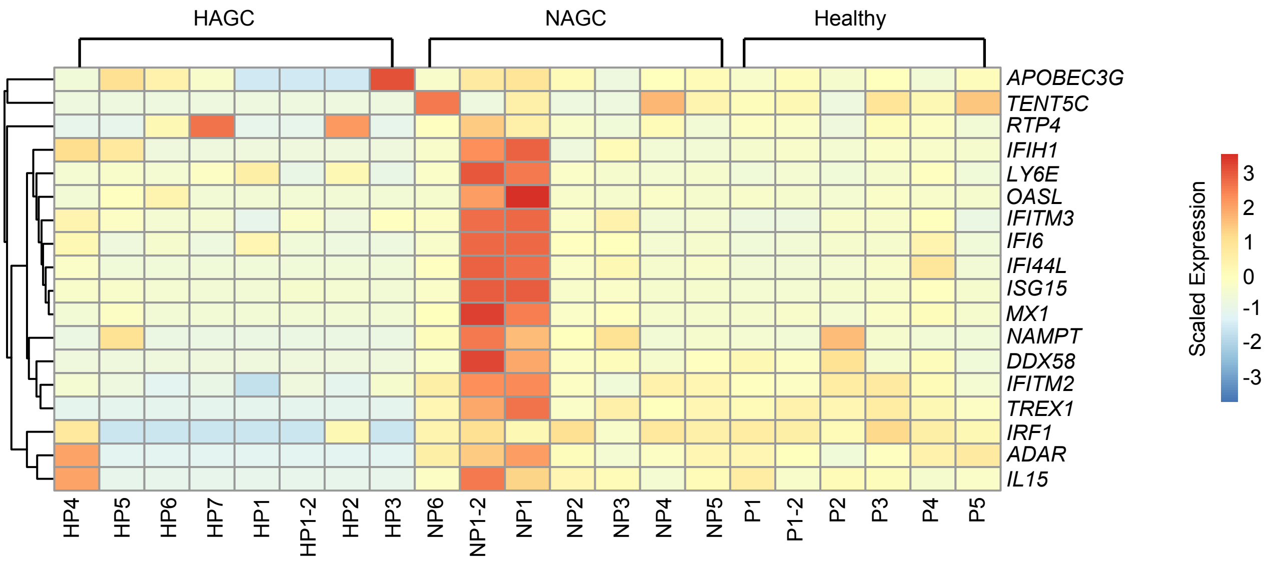
HAGC
NAGC
Healthy
APOBEC3G
TENT5C
RTP4
IFIH1
LY6E
OASL
IFITM3
IFI6
IFI44L
ISG15
MX1
NAMPT
DDX58
IFITM2
TREX1
IRF1
ADAR
IL15
HP4
HP5
HP6
HP7
HP1
HP1-2
HP2
HP3
NP6
NP1-2
NP1
NP2
NP3
NP4
NP5
P1
P1-2
P2
P3
P4
P5
Scaled Expression
3
2
1
0
-1
-2
-3

Figure S4

C

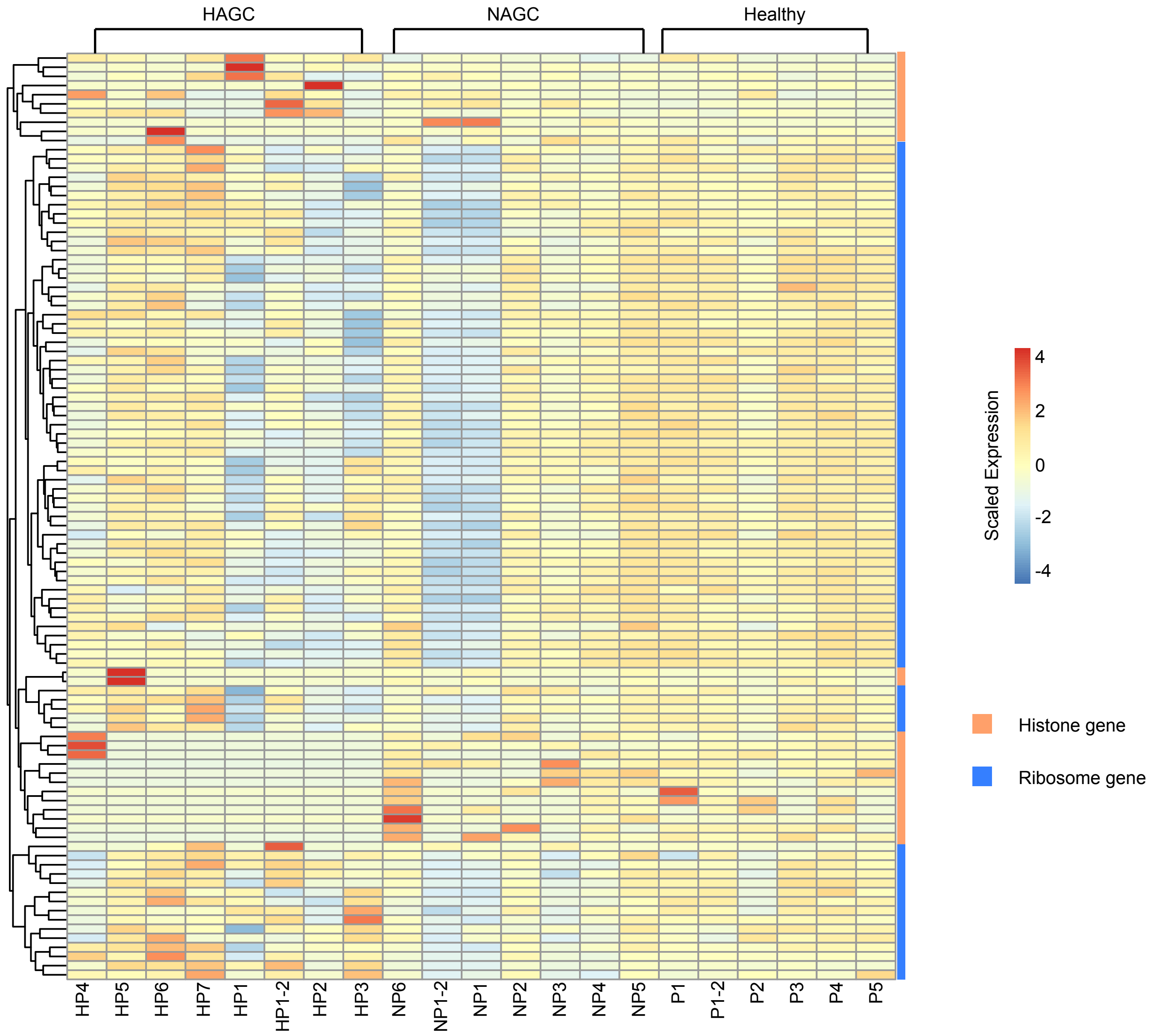

HAGC
NAGC
Healthy
HP4
HP5
HP6
HP7
HP1
HP1-2
HP2
HP3
NP6
NP1-2
NP1
NP2
NP3
NP4
NP5
P1
P1-2
P2
P3
P4
P5
Scaled Expression
4
2
0
-2
-4
Histone gene
Ribosome gene

Figure S5

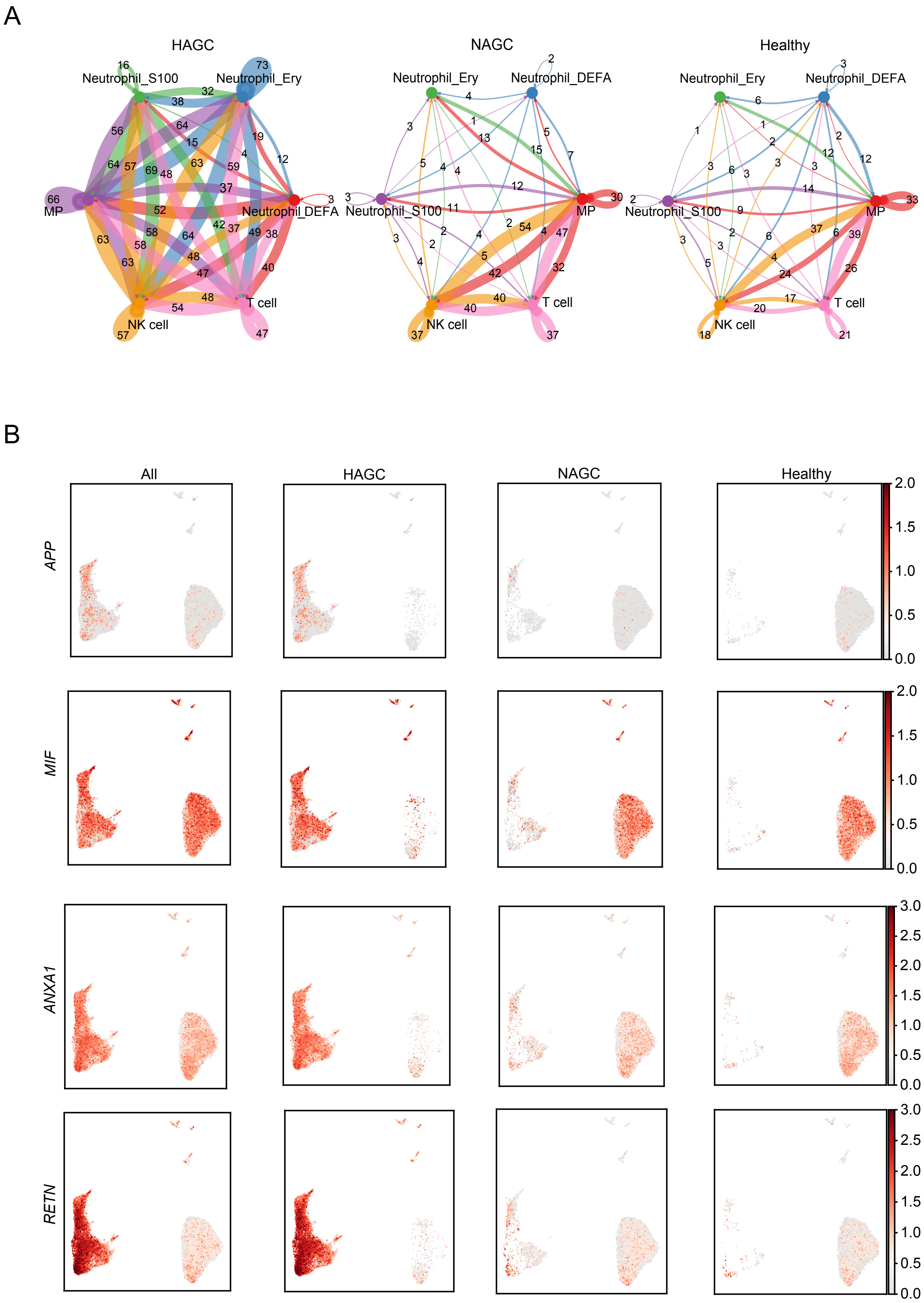

A
HAGC
NAGC
Healthy
Neutrophil_S100
Neutrophil_Ery
Neutrophil_DEFA
MP
T cell
NK cell
B
All
HAGC
NAGC
Healthy
APP
MIF
ANXA1
RETN

Figure S5

C

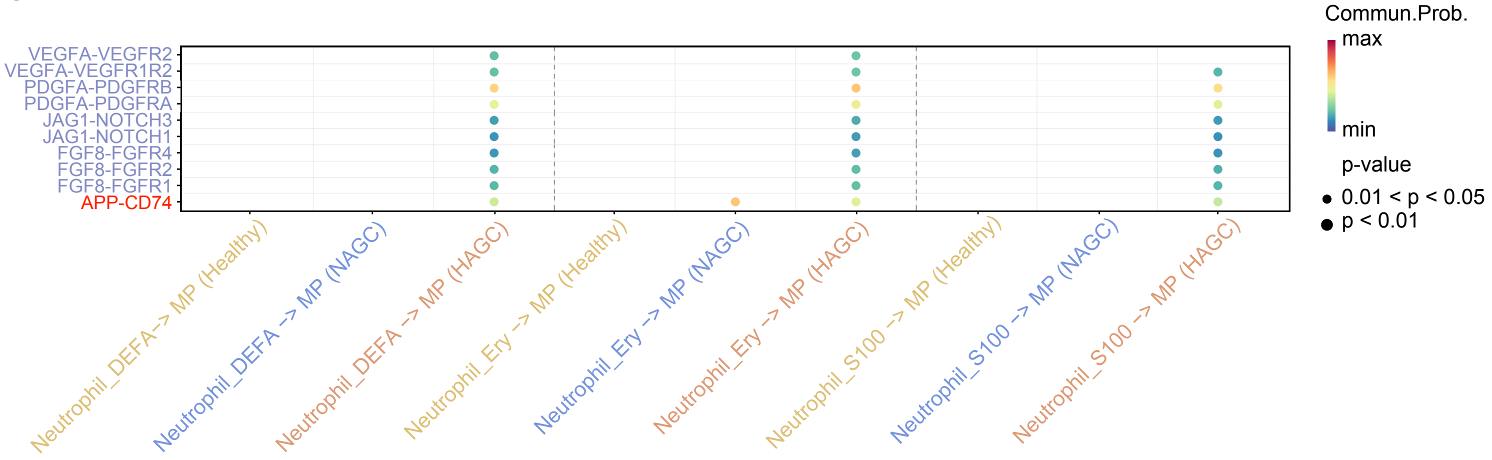

D

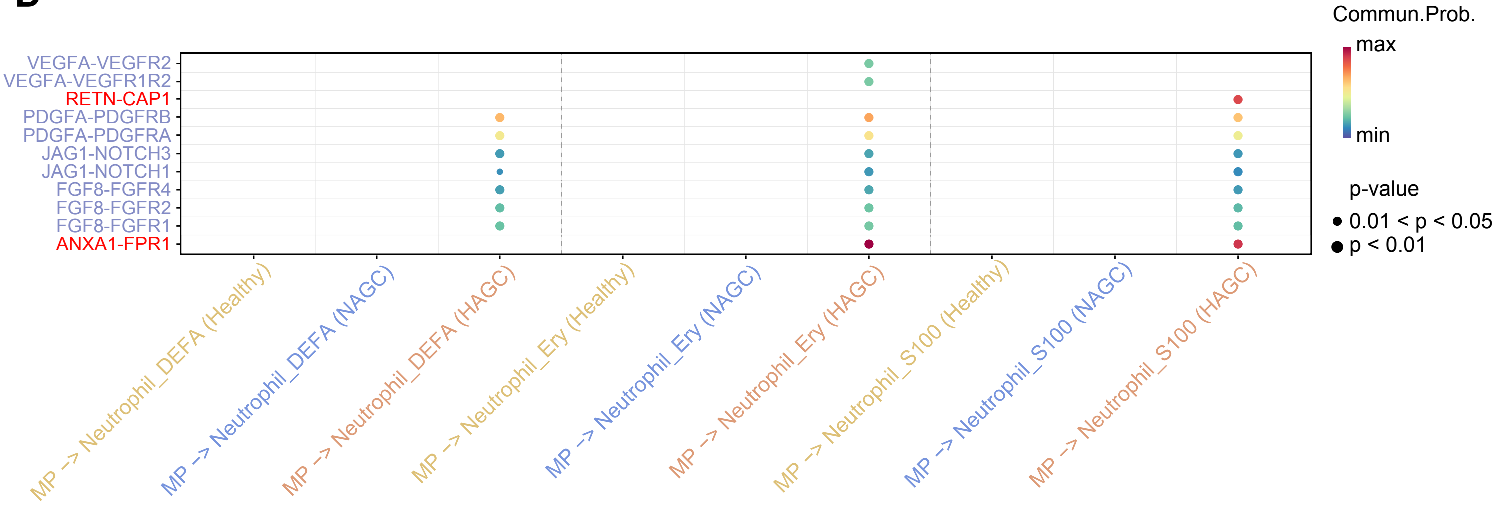

E

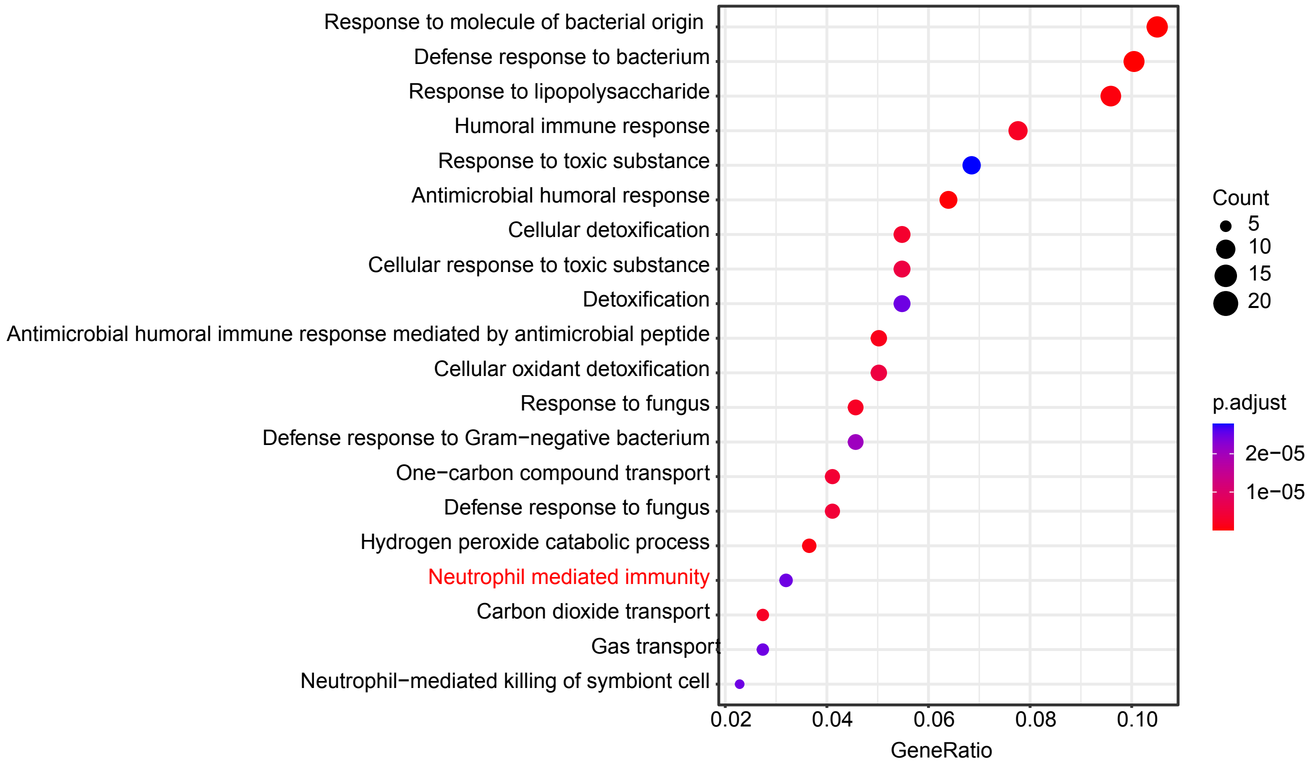

Figure S6

A

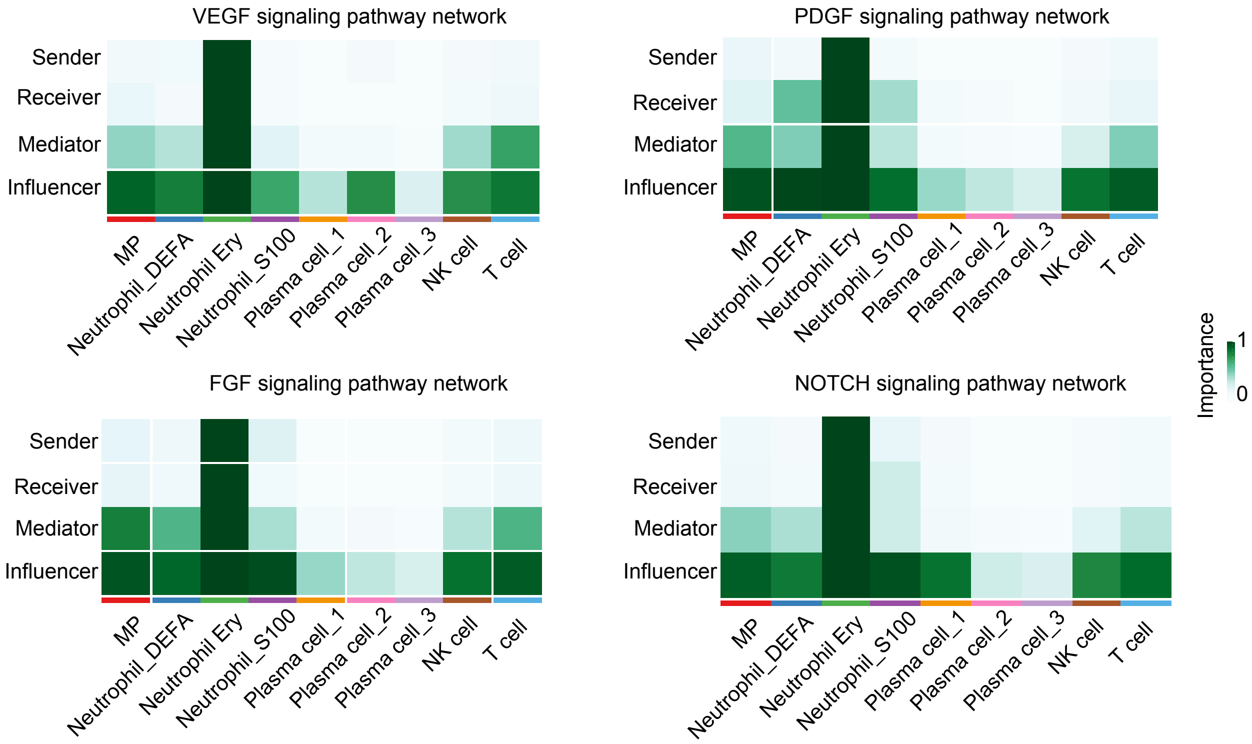

B

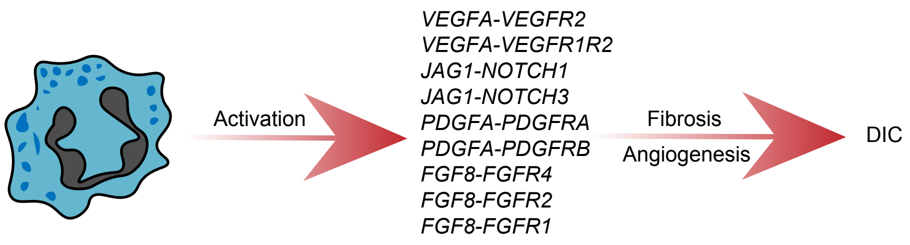

C

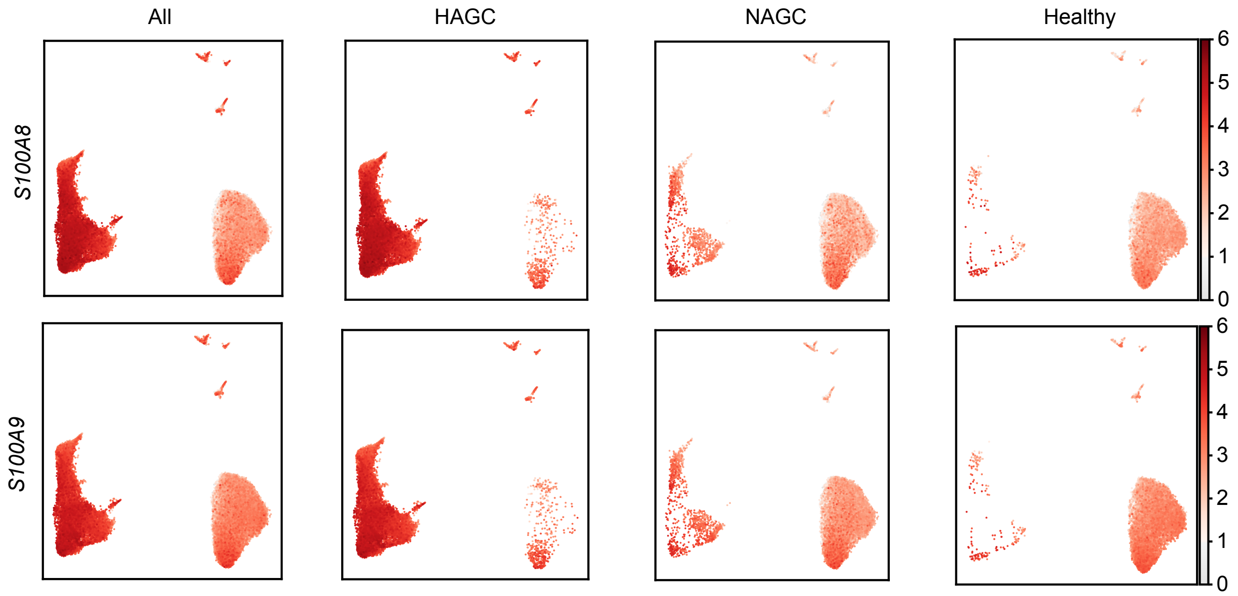

Supplement: Supplementary file 1 — Supplementary Figure S1. Single cell RNA‐seq data analysis of PBMCs in HAGC patients, NAGC patients and healthy controls. (A) Violin plots illustrating the average number of genes detected per patient sample (left) and the average number of counts per cell for each patient sample (right). ‘HP’ represents HAGC patients, ‘NP’ represents NAGC patients and ‘P’ represents healthy donors. (B) Pie chart displaying the distribution of cells within each group among all the successfully filtered cells. (C) UMAP visualization depicting the expression pattern of canonical marker genes within each cluster, with color‐coded representation based on the expression of selected marker genes. The colour scheme indicates the expression level of the respective genes. Erythroblasts (HBB, HBA1), neutrophils (DEFA3, DEFA1, S100A9, S100A8), monocytes (LYZ, CD14), T cells (CD3D, CD3E), NK cells (CCL5 and NKG7), and B cells (JCHAIN, IGHA1, IGLC2, IGLL5). (D) Scaled bar chart showing the proportion of cell types in each sample. Supplementary Figure S2. Characteristics of neutrophils in HAGC. (A) Split UMAP visualization displaying the distribution of three clusters (Neutrophil_DEFA, Neutrophil_Ery, and Neutrophil_S100) within neutrophils, colour coded by the origin of each cluster. (B) UMAP representation of the expression patterns of canonical marker genes within each subcluster (Figure 2A), with color‐coding based on the expression of selected genes. The colour scheme reflects the expression level of each gene. Group 0 (HBA2, HBB), Group 1 (LTF, CAMP), Group 2 (IFI30, AC007192.1), Group 3/5 (DEFA1, DEFA1B, DEFA3), Group 4 (L1PA6, TMSB10), and Group 6 (ELANE, PRTN3, MPO). (C) Boxplot demonstrating the predicted differentiation score of neutrophil subclusters in HAGC, NAGC, and healthy individuals by CytoTRACE. (D) scVelo RNA velocity estimating the interrelationship between neutrophil subclusters. The velocity fields were projected onto the UMAP distribution. (E) UMAP representation of neutro [file CPR-57-e13591-s004.pdf]
